# An Isoform of the Oncogenic Splice Variant AIMP2-DX2 Detected by a Novel Monoclonal Antibody

**DOI:** 10.3390/biom10060820

**Published:** 2020-05-27

**Authors:** Dae Gyu Kim, Thi Thu Ha Nguyen, Nam Hoon Kwon, Junsik Sung, Semi Lim, Eun-Joo Kang, Jihye Lee, Woo Young Seo, Arum Kim, Yoon Soo Chang, Hyunbo Shim, Sunghoon Kim

**Affiliations:** 1 Medicinal Bioconvergence Research Center, College of Pharmacy and College of Medicine, Gangnam Severance Hospital, Yonsei University, Incheon 21983, Korea; kimeorb@hanmail.net (D.G.K.); nanaromi@biocon.snu.ac.kr (N.H.K.); jsung7028@icloud.com (J.S.); smlim226@naver.com (S.L.); dmswn3328@hanmail.net (E.-J.K.); jh_lee@curebio.co.kr (J.L.); swy435@gmail.com (W.Y.S.); 2 Department of Life Science, Ewha Womans University, 52 Ewhayeodae-gil, Seodaemun-gu, Seoul 03760, Korea; hanguyen15490@gmail.com; 3 Department of Internal Medicine, Yonsei University College of Medicine, 63 Gil-20, Eonju-ro, Gangnam-gu, Seoul 06229, Korea; karmy318@yuhs.ac (A.K.); yschang@yuhs.ac (Y.S.C.)

**Keywords:** antibody, splice variant, AIMP2-DX2, phage display, diagnostic marker

## Abstract

AIMP2-DX2, an exon 2-deleted splice variant of AIMP2 (aminoacyl-tRNA synthetase-interacting multifunctional protein 2), is highly expressed in lung cancer and involved in tumor progression in vivo. Oncogenic function of AIMP2-DX2 and its correlation with poor prognosis of cancer patients have been well established; however, the application of this potentially important biomarker to cancer research and diagnosis has been hampered by a lack of antibodies specific for the splice variant, possibly due to the poor immunogenicity and/or stability of AIMP2-DX2. In this study a monoclonal antibody, H5, that specifically recognizes AIMP2-DX2 and its isoforms was generated via rabbit immunization and phage display techniques, using a short peptide corresponding to the exon 1/3 junction sequence as an antigen. Furthermore, based on mutagenesis, limited cleavage, and mass spectrometry studies, it is also suggested that the endogenous isoform of AIMP2-DX2 recognized by H5 is produced by proteolytic cleavage of 33 amino acids from N-terminus and is capable of inducing cell proliferation similarly to the uncleaved protein. H5 monoclonal antibody is applicable to enzyme-linked immunosorbent assay, immunoblot, immunofluorescence, and immunohistochemistry, and expected to be a valuable tool for detecting AIMP2-DX2 with high sensitivity and specificity for research and diagnostic purposes.

## 1. Introduction

Since the advent of targeted therapies and precision medicine, companion diagnostics have become an essential tool for patient stratification and selection [1]. Most of all, cancer treatments driven by biomarkers of clinical relevance have made a big breakthrough for various cancers by providing personalized treatment options and better outcome for patients. For example, molecular diagnoses for mutations in epidermal growth factor receptor gene (*EGFR*) and for the anaplastic lymphoma kinase gene (*ALK*) rearrangements have opened new therapeutic opportunities for EGFR-positive solid tumors and ALK-positive non-small cell lung cancer, respectively; however, despite the recent attempts to discover novel targets from the uncharacterized genes of human genome, there are still over 43% cancer patients for whom no biomarkers or targeted therapies are available [2].

ARS-interacting multi-functional protein 2 (AIMP2) is a scaffolding protein that forms a part of the multi-tRNA synthetase complex (MSC) involved in protein translation. When released from MSC, AIMP2 also functions as an anti-proliferative factor via TGF-β, TNF-α, UV, and Wnt signaling [3,4,5,6,7,8]. In contrast to AIMP2, AIMP2-DX2 (hereafter referred to as DX2), an alternative splicing variant of AIMP2 lacking exon 2, shows oncogenic activity, competitively interfering with the tumor suppressive function of AIMP2 upon various signaling [9,10]. DX2 is inducible by the carcinogen benzopyrene which causes mutations in and the skipping of exon 2 [10], and inhibits cancer cell apoptosis by directly interacting with p14/ARF [11]. High expression of DX2 was significantly associated with the poor prognosis of lung cancer patients and the cancer progression, and knockdown of DX2 using siRNA or a small molecule significantly reduced tumor progression in in vivo [10,12], suggesting that DX2 can be a promising cancer target and a diagnostic as well as prognostic marker for anti-cancer therapies. Other than DX2, no genetic alterations of AIMP2 is known to be associated with human cancers, although a homozygotic nonsense mutation of AIMP2 has been reported to be associated with hypomyelinating leukodystrophy-17, an autosomal recessive neurodevelopmental disorder [13].

Keeping up with the drug development efforts targeting DX2 [10,11], many attempts have been made toward the development of monoclonal antibodies (mAbs) specific to DX2 that can be used as a diagnostic agent. Currently, clone #324 is the only antibody that can be used during immunoblotting to detect DX2. Clone #324 is a mAb obtained by immunization of a mouse with AIMP2 as an immunogen. Although the major protein recognized by clone #324 during immunoblotting is AIMP2, it additionally detected DX2 along with several unidentified bands. So far, all the attempts to develop a DX2-specific antibody by immunization or bio-panning using DX2 peptides or proteins as an antigen have failed. In this study, we report the development of a new anti-DX2 mAb, H5, by rabbit immunization followed by phage display selection. We also suggest that the endogenous DX2 isoform recognized by H5 lacks 33 amino acids (aa) in the N-terminus and is a functional surrogate for DX2. The possible application of the DX2-specific antibody to immunohistochemistry (IHC), immunofluorescence (IF), and enzyme-linked immunosorbent assay (ELISA) indicated the potential utility of this antibody as a tool for research as well as the identification of cancer patients with high levels of DX2.

## 2. Materials and Methods

### 2.1. Ethics Statements

All animal studies involving rabbit immunization, euthanasia, and organ harvesting were conducted by AbClon Inc. (Seoul, Korea) in accordance with the guidelines of the National Institute of Health (NIH) “Guide for Care and Use of Animals” and an approved protocol received by the company’s Institution Animal Care and Use Committee. For IHC staining, non-small cell lung cancer (NSCLC) tissues from patients who underwent lung resection between 2000 and 2008 and had submitted written consent providing the residual samples were randomly selected from the institutional tissue archives. The use of tumor tissue was approved by the Institutional Review Board (IRB) of Gangnam Severance Hospital (IRB #3-2014-0299) and was carried out in compliance with the Declaration of Helsinki and Korean Good Clinical Practice guidelines.

### 2.2. Rabbit Immunization, mRNA Extraction, and cDNA Synthesis

A synthetic peptide corresponding to the sequence spanning the exon 1–3 junction of DX2, GHVQDYGALKDC (C-terminal Cys added for conjugation reaction), was conjugated to keyhole limpet hemocyanin (KLH). A total of 200 µg KLH-conjugated antigen peptide was dissolved in 500 µL phosphate-buffered saline (PBS, pH 7.4) and mixed with 500 µg of adjuvant. Two rabbits were subcutaneously injected with 1 mL of the antigen/adjuvant mixture at 7–10 day intervals. An aliquot of blood was taken from each animal 1 week after each immunization and titrated by ELISA to determine the presence of antigen-specific antibodies. Following the final immunization, the rabbits were exsanguinated, and the spleens and blood sera were harvested. After weighing, splenic tissues were placed in TRI-reagent^®^ (Sigma-Aldrich) and homogenized. Additional 15 mL of TRI-reagent was added and the homogenate was incubated for 5 min at room temperature (RT). Phase separation was carried out by addition of 1.5 mL of BCP (1-bromo-3-chloropropane). The homogenate was thoroughly mixed for 15 Sec, incubated for 15 min at RT, and centrifuged for 15 min at 12,000 rpm. The supernatant was carefully transferred to a clean tube, avoiding contamination by the fatty layer and pellet separated on the bottom of the tube, and mixed with 7.5 mL of isopropanol for RNA precipitation. The tubes were vortexed and incubated at RT for 10 min, and centrifuged at 12,000 rpm for 10 min at 4 °C to pellet the RNA. The supernatant was removed, and the pellet was washed with 10 mL of 70% ethanol and incubated on ice for 5 min. After centrifugation at 12,000 rpm for 10 min at 4 °C, ethanol was discarded and the pellets were air-dried for 3 min and resuspended in 1 mL of nuclease-free water. Total splenic RNA was further purified by lithium chloride (LiCl) precipitation [14]. Briefly, RNA was precipitated by adding 53 µL of 7.5 M LiCl to 500 µL of RNA solution, incubating the mixture on ice for 2 h, and centrifuging at 12,000 rpm for 30 min. The pellet was dissolved in 500 µL nuclease-free water, and the RNA was LiCl-precipitated again as above. The pellet was then dissolved in nuclease-free water, and ethanol-precipitated (0.1 volume of 3 M sodium acetate (pH 5.2) and 2.2 volume of ethanol, incubated on ice for 1 h). The precipitated RNA was centrifuged and dissolved in 200 µL nuclease-free water. First-strand cDNA was synthesized using the reverse transcription-polymerase chain reaction (RT-PCR) kit (Thermo Fischer scientific). For complementary DNA (cDNA) synthesis, 1 µL of total RNA was mixed with 1 µL of oligo (dT)_18_ primer, 1 µL of 10 mM dNTP mix, 4 µL of 5× RT buffer, 1 µL of Maxima H minus enzyme mix, and water to a final volume of 20 µL. The reaction was performed for 30 min at 50 °C and terminated by heating at 85 °C for 5 min. The first-strand cDNA was immediately used for PCR amplification of antibody variable domain genes.

### 2.3. Generation of Rabbit Fab (Fragment Antigen-Binding)

First-strand cDNA derived from the rabbit spleen was used as the template to amplify VH (heavy chain variable) and VL (light chain variable) domain genes, and pComb3X-TT phagemid vector [15] was used for the amplification of human C_κ_ (constant kappa chain) and CH1 (first constant heavy chain) domains to construct Fab antibody library. Primers used for the amplification of individual domains and their combinations are listed on Tables S1 and S2, respectively. PCR using *Taq* polymerase for the amplification of the variable and the constant domains (~350 bp each) was performed under the following condition: 94 °C for 2 min; 30 cycles of 94 °C for 30 Sec, 56 °C for 30 Sec, and 72 °C for 30 Sec; followed by 72 °C for 7 min. Then, each gene fragment was purified using gel extraction kit (Qiagen) after the PCR reaction products were separated by 1% agarose gel electrophoresis. In the second round of PCR, each of the heavy and the light chain variable fragments was joined to the respective constant domain by overlap extension PCR using *Taq* polymerase (New England Biolabs). The PCR program was set under the following condition: 94 °C for 2 min; 25 cycles of 94 °C for 30 Sec, 56 °C for 30 Sec, and 72 °C for 1 min; followed by 72 °C for 10 min. Primers for overlap extension PCR are listed in Table S3. Approximate sizes of the heavy chain VH-CH1 fragment (Fd) and light chain fragment (LC) were 750 bp and 800 bp, respectively. Finally, the Fd and LC products were joined by PCR again using *Taq* polymerase and RSC-SF/dpseq primers. The PCR program was as follows: 94 °C for 2 min; 25 cycles of 94 °C for 30 Sec, 56 °C for 30 Sec, and 72 °C for 1.5 min; followed 72 °C for 10 min. Approximate size of final Fab product was 1500 bp.

### 2.4. Construction of Fab Library

The rabbit-human chimeric Fab library was cloned into pComb3X phagemid vector. First, the vector and the Fab DNA were digested with SfiI restriction enzyme (New England Biolabs). For digestion of DNA, 15 µg of purified PCR product or vector DNA was mixed with 50 units of SfiI, 20 µL of 10× reaction buffer, and water to a final volume of 200 µL. Digestion mixtures were incubated at 50 °C for 16 h followed by ethanol precipitation overnight at −20 °C. The precipitated DNA was centrifuged and dissolved in 100 µL nuclease-free water, and purified by agarose gel electrophoresis. Digested vector and Fab DNA fragments were ligated overnight (2 μg vector DNA and 3 μg insert DNA (~1:3 molar ratio) in 100 µL total volume) and ethanol precipitated. After centrifugation, the ligated DNA was dissolved in 10 µL of nuclease-free water and transformed to *Escherichia coli* (*E. coli*) ER2537 cells by electroporation. Transformed bacteria were plated on LB (Luria-Bertani broth)-ampicillin plates with 2% (w/v) glucose and incubated overnight. Next day, the bacterial growth was scraped and resuspended in 5 mL SB (Super Broth) medium, 15% (v/v, final concentration) glycerol was added, and 1 mL aliquots of the bacterial suspension were kept at −80 °C. The immune Fab phage library was rescued from the bacterial stock as previously reported [16].

### 2.5. Panning of Phage Displayed Antibody Library

The phage displayed Fab library was panned against DX2-BSA antigen (exon 1/3 junction sequence peptide conjugated to bovine serum albumin [BSA]). For each round of selection, 3 µg of antigen in 1 mL of PBS was coated on an immunotube (NUNC 470319, Thermo Fisher Scientific, Waltham, MA, USA). The tube was coated for 1 h at 37 °C and subsequently blocked by filling the tube with 3% skim milk in PBST (PBS with 0.05% Tween 20, pH 7.4) for 1 h at RT. The blocking solution was removed and the phage library (10^12^ colony forming units in 1 mL of the blocking solution) was added to the antigen-coated immunotube. The tube was then incubated with shaking at 37 °C for 2 h, and the unbound phages were washed out three times with PBST. The bound phages were eluted with 1 mL of 100 mM TEA (triethanolamine) for 10 min at RT, and the eluted phage solution was neutralized with 500 µL of 1 M Tris-HCl (pH 7.4). The neutralized phages were applied to 8.5 mL mid-log phase ER2537 and incubated for 1 h at 37 °C with shaking at 120 rpm. Infected ER2537 cells were plated on ampicillin-LB agarose plate with 2% (w/v) glucose and grown overnight at 37 °C. The next day, the bacteria were harvested and phage library was rescued. Identical panning steps were repeated three times for the enrichment of antibody specific to the target peptide.

### 2.6. Screening for Phage Library Clones

Following three rounds of panning selection, output clones were screened by ELISA to identify specific target binders. Individual bacterial colonies were grown in 96-well plates containing 200 µL SB per well supplemented with 100 µg/mL ampicillin at 37 °C with vigorous shaking. After 3–4 h, when most wells became turbid, 1 mM final concentration of IPTG (isopropyl β-D-1-thiogalactopyranoside) was added to each well, and the induced cells were grown at 30 °C overnight with shaking. Prior to induction, replicate plates were prepared using a 96-pin plate replicator (Boekel Scientific, Feasterville-Trevose, PA, USA). Next day, IPTG-induced cells were spun down at 3500× *g* for 15 min and supernatants were discarded. Periplasmic extracts (PPEs) were obtained by resuspending the cell pellet in 60 μL of cold 1× TES buffer (30 mM Tris-HCl, 1 mM EDTA (ethylenediaminetetraacetic acid), 20% sucrose; pH 8.0), subsequently adding 90 μL of cold 0.2× TES buffer, and incubating on ice for 30 min. PPEs were applied to ELISA plates (25 µL per well) previously coated with either 100 ng/well of the antigen or PBS (blank control) and blocked with 3% skim milk-PBST. Plates were incubated for 1 h at RT, washed three times with PBST, and 25 µL per well of HRP (horseradish peroxidase)-conjugated anti-HA tag antibody (Santa Cruz Biotechnology clone F-7, 1:3000 dilution) was added to each well. After 1 h binding, plates were washed three times with PBST and 25 µL of TMB (3,3′,5,5′-tetramethylbenzidine) solution was added to each well. As blue color developed, 25 µL of 1 M sulfuric acid per well was added to stop the reaction. Absorbance was read at 450 nm in a plate reader. 

### 2.7. Fab and Immunoglobulin G (IgG) Purification

To produce Fab, 20 mL of SB-ampicillin medium was inoculated with 5 µL of *E. coli* stock of the clone of interest from the replicate plate (see above). When the culture became turbid (absorbance at 600 nm > 0.5), 1 mM IPTG was added and the cells were further cultured overnight at 30 °C. The next day, cells were centrifuged and resuspended in 1 mL of cold 1× TES. Subsequently, 1.5 mL of cold 0.2× TES was added and mixed well, and the cell suspension was incubated on ice for 30 min. After centrifugation, the supernatant was transferred to a 15 mL conical tube, 100 µL of Ni-NTA (Ni^2+^-coordinated nitrilotriacetic acid) agarose beads was added, and the mixture was incubated at 4 °C with slow rotation for 1 h. The tube was centrifuged, supernatant was discarded, and the beads were washed twice with PBS containing 5 mM imidazole. Fab protein was eluted from the beads in 100 µL fractions using PBS containing 200 mM imidazole (pH 7.4). H5 Fab isolated from the library was also reformatted to human and rabbit IgGs. Heavy and light chain constant regions with an upstream signal sequence for protein secretion had previously been cloned in multiple cloning sites of the dual-promoter plasmid pVITRO1 (Invivogen, San Diego, CA, USA). VH and VL genes of H5 were amplified by PCR and serially cloned to this modified pVITRO1 vector harboring human or rabbit IgG constant region genes. The resulting human H5 IgG (hH5) or rabbit H5 IgG (rH5)-expression vector was transfected to 293F suspension cell line. Briefly, 100 µg of polyethyleneimine (PEI, linear, MW ~25,000; Polysciences Inc., Warrington, PA, USA) in 2.5 mL of sterile PBS was added to 100 µg of the vector DNA in 2.5 mL of sterile PBS. The mixture was incubated for 15 min at RT, and added dropwise to 50 mL of 293F cell culture at 2 × 10^6^ cells/mL. Next day, 50 mL of fresh Freestyle 293 medium (Thermo Fisher Scientific, Waltham, MA, USA) was added to the cells, and the flask was incubated with shaking (~120 rpm) for 5 days at 37 °C under 8% CO_2_. After 5 days, cells were centrifuged, and H5 IgG was purified from the supernatant using protein A-agarose column chromatography.

### 2.8. Cell Culture

H460, HCC827, HCC15, H2108, DLD-1, HCC1419, SU8686, MKN74, NCC59, MKN45, and CHO-K1 cells were cultured in RPMI medium. SW620, MDA-MB 231, MIA-Paca3, BxPc3, DU145, and HEK293T cells were cultured in Dulbecco’s modified Eagle medium (DMEM). All of the cell lines were cultured in medium supplemented with 10% FBS and 1% penicillin/streptomycin at 37 °C in 5% CO_2_ incubator. Freestyle 293 cells for antibody purification were cultured with shaking at 120 rpm in Freestyle 293 Expression Medium at 37 °C under 8% CO_2_.

### 2.9. Plasmids, siRNA, and Transfection

Three different siRNAs were designed and purchased from Oligo Center, ST Pharm Co. Ltd. (Gyeonggi, Korea) The sequences of siRNAs against DX2 were as follows: siRNA-1, CUGGCCACGUGCAGGAUUA; siRNA-2, GGAACAUUGCACGUUUCUU; and siRNA-3, GCUGUCAACGCAACCCUUA. Strep-tagged full-size DX2 (DX2-F), DX2_2-251_, DX2_23-251_, and DX2_34-251_ were cloned in pEXPR IBA105 vector. Tag free DX2 gene was inserted into pcDNA3.1(+) vector using NheI/XhoI sites. To generate N-terminal deleted DX2 isoforms, an initiation codon (ATG) was inserted in front of each DX2 isoform sequence. Each of the siRNAs against DX2 and for negative control (Invitrogen) was transfected into H460 cells using Lipofectamine 2000 (Invitrogen), according to the manufacturer’s protocol. HEK293T cells and CHO-K1 cells were transfected with tag-free DX2 or Strep-tagged DX2 using Turbofect (Thermo Fisher Scientific, Waltham, MA, USA) reagent. The cells were incubated for at least 20 h prior to experiments.

### 2.10. Protein Purification

Tag-free DX2 protein was purified as previously described [17]. C-terminal his-tagged DX2_34-251_ and AIMP2_34-320_ genes were cloned into pET-28a vector and overexpressed in *E. coli* BL21(DE3) by induction with 0.5 mM IPTG. After centrifugation, cells were resuspended and sonicated in buffer (35 mM imidazole, 500 mM NaCl, and 20 mM Tris-HCl (pH 7.5)) supplemented with PMSF (phenylmethylsulfonyl fluoride) and protease inhibitor cocktail (Calbiochem, San Diego, CA, USA). After centrifugation, the supernatant was loaded onto a HiTrap chelating HP column (GE Healthcare, Chicago, IL, USA) pre-equilibrated with the same buffer without the protease inhibitors. The column was washed with the equilibration buffer, and the protein was eluted with the elution buffer (1 M imidazole, 500 mM NaCl, and 20 mM Tris-HCl (pH 7.5)). The partially purified proteins were further subjected to size exclusion (HiLoadl 6/600 Superdex75, GE Healthcare, Chicago, IL, USA) and cation exchange (HiTrap Sp HP) chromatographies. 

### 2.11. Limited Proteolysis with Trypsin

Purified DX2 protein and trypsin (Promega, Madison, WI, USA) was mixed in reaction buffer containing 20 mM Tris/HCl (pH 7.5), 150 mM NaCl, 1 mM dithiothreitol (DTT), and 10% glycerol. Reaction ratio of trypsin to DX2 protein was 1:1000 and mixture was incubated for 1 min at 25 °C. Reaction was stopped by adding SDS (sodium dodecyl sulfate) sample buffer and boiled for 10 min at 100 °C to halt enzyme reaction. Samples were subjected to liquid chromatography-mass spectrometry (LC-MS) analysis or Western blotting after SDS-PAGE (sodium dodecyl sulfate-polyacrylamide gel electrophoresis).

### 2.12. LC-MS/MS and Data Analysis

DX2 protein was digested in-gel by trypsin as previously reported [18]. Peptide sample was analyzed on a LTQ-OrbitrapVelos (Thermo Fisher Scientific, Waltham, MA, USA) connected to an Easy-nano LC II system (Thermo Fisher Scientific, Waltham, MA, USA). The dried peptide sample was resuspended in 70 µL of 0.1% formic acid, and an aliquot (7 μL) was injected to a reverse-phase peptide trap EASY-Column (L 2 cm, ID 100 μm, 5 μm, 120 Å, ReproSil-Pur C18-AQ, Thermo Fisher Scientific, Waltham, MA, USA) and a reversed-phase analytical EASY-Column (L 10 cm, ID 75 μm, 3 μm, 120 Å, ReproSil-Pur C18-AQ, Thermo Fisher Scientific, Waltham, MA, USA), and electrospray ionization was subsequently performed using a 30 μm (internal diameter) nano-bore stainless steel online emitter (Thermo Fisher Scientific, Waltham, MA, USA). The duration of total LC gradient was 60 min. The peptides were eluted in a linear gradient of 10%–40% buffer B (0.1% formic acid in acetonitrile) and 90%–60% buffer A (0.1% formic acid in H_2_O) over 40 min and a flow rate of 300 nL/min. The temperature and voltage applied to the capillary was 275 °C and 1.9 V, respectively. All data were acquired with the mass spectrometer operating in automatic data-dependent switching mode. The MS survey was scanned from 350 to 2000 m/z with resolution set to 100,000. All MS/MS samples were analyzed using Sequest (XCorr Only, Thermo Fisher Scientific, Waltham, MA, USA, version v.27, rev. 11), X! Tandem (The GPM, thegpm.org; version CYCLONE (2010.12.01.1)) and the human sequence database (Uniprot 2014). Search parameters were set as follows: Full digestion using trypsin/Lys-C (after KR/-) with up to two missed cleavages, and precursor and fragment mass tolerances of 25 ppm and 1.0 Da, respectively. Each processed data was subsequently transformed to *.sf file with Scaffold 4 Q + S program (Proteome Software Inc., Portland, OR, USA, version 4.6.1). Scaffold program was used to validate MS/MS based peptide and protein identifications and to process the quantitative analysis. Peptide identifications were accepted if they could be established at greater than 80.0% probability by the Peptide Prophet algorithm [19] with Scaffold delta-mass correction. Protein identifications were accepted if they could be established at greater than 80.0% probability and contained at least two identified peptides. Protein probabilities were assigned by the Protein Prophet algorithm [20].

### 2.13. Surface Plasmon Resonance (SPR)

Surface Plasmon Resonance analysis were performed using a Biacore T200 (GE Healthcare, Chicago, IL, USA) equipped with a Series S sensor chip CM5 (GE Healthcare, Chicago, IL, USA). Immobilization was performed using amine coupling kit (GE Healthcare, Chicago, IL, USA) with PBS as a running buffer. Flow cells were activated with a 7 min pulse of a 1:1 mixture of EDC (1-ethyl-3-(3-dimethylaminopropyl)-carbodiimide hydrochloride) and NHS (*N*-hydroxysuccinimide) according to the manufacturer’s instructions. hH5 mAb was diluted in 10 mM sodium acetate (pH 5.0) and immobilized to the chip, giving a surface density of 3320 response units (RU). Flow cells were then blocked with a 7 min pulse of 1 M ethanolamine-HCl (pH 8.5). For the interaction analysis, epitope peptide was diluted in PBS buffer supplemented with NSB (non-specific binding) reducer (GE Healthcare) and a two-fold serial dilution series was created to generate a concentration range of 0.02 to 1 µM. Proteins (DX2-F, DX2_34-251_, and AIMP2_34-320_) were diluted with 10 mM sodium acetate (pH 5.0) and immobilized to a Series S sensor chip CM5 at an immobilization levels of 590 RU. hH5 or rH5 mAb was diluted in PBS buffer with NSB reducer producing a 2-fold serial dilution series with a concentration range from 0.04 to 1 µM. After each binding cycle, regeneration solution (10 mM glycine-HCl, pH 1.5) was injected to remove any non-covalently bound protein. The binding data was fitted into a 1:1 binding model in Biacore T200 Evaluation software v2.0 (GE Healthcare, Chicago, IL, USA).

### 2.14. Enzyme-Linked Immunosorbent Assay (ELISA)

Each protein was diluted in 0.05 M sodium carbonate buffer (pH 9.6) to a final concentration of 1.5 ng/µL, and 100 µL/well was added to 96-well ELISA plates (Thermo Fisher Scientific, Waltham, MA, USA) and incubated overnight at 4 °C. After washing three times with 1× PBST, wells were blocked with 1% BSA in PBST for 1 h at RT. The plates were incubated with serially diluted hH5 or rH5 mAb for 1 h at RT, washed three times with PBST, and incubated with anti-human or anti-rabbit IgG secondary antibody (1:10,000 dilution). After 1 h binding, plates were washed three times with PBST and 100 µL of TMB substrate solution was added to each well. As blue color developed 10 min later, 1 M sulfuric acid was added (50 µL/well) to stop the reaction, and absorbance was read at 450 nm in a plate reader. The data were fitted to the 4-parameter logistic curve model using GraphPad Prism (GraphPad Software, San Diego, CA, USA).

### 2.15. Immunoprecipitation

H460 or HEK293T cells were rinsed with cold PBS, lysed in lysis buffer (50 mM Tris-HCl, 250 mM NaCl, 0.5% NP-40, and 5 mM EDTA) for 20 min at 4 °C, and centrifuged at 13,000 rpm for 30 min at 4 °C. The supernatant was incubated with rH5 mAb overnight. Protein A agarose beads (Invitrogen, Carlsbad, CA, USA) were added, and the mixture was incubated with rotation at 4 °C for 2 h. The beads were washed with cold lysis buffer five times, and the precipitates were subjected to SDS-PAGE.

### 2.16. Immunoblotting

Cells were lysed in cold RIPA (radioimmunoprecipitation assay) lysis buffer (25 mM Tris-HCl [pH 7.4], 125 M NaCl, 0.5% NP-40, 0.25% sodium deoxycholate, and 0.5% SDS, supplemented with protease inhibitors) for 20 min at 4 °C. After centrifugation at 13,000 rpm for 30 min at 4 °C, the supernatant proteins were quantified by Bradford assay (BioRad, Hercules, CA, USA). Proteins were separated by 10%–15% SDS-PAGE and transferred to 0.45 µm nitrocellulose or PVDF (polyvinylidene difluoride) membrane. After blocking, the membrane was incubated with primary antibody solution overnight at 4 °C, followed by washing three times with PBST and binding by HRP-conjugated secondary antibody for 1 h at RT. The membrane was washed three times with PBST, and subjected to luminescence detection using ECL (enhanced chemiluminescence) solution (GE Healthcare, Chicago, IL, USA). For the competition binding study, epitope peptide (100 µg/mL) was added during primary antibody (H5 Fab) binding. ImageJ software was used for the quantification of the detected band.

### 2.17. Immunofluorescence Staining

CHO-K1 cells were transfected with the DX2 or AIMP2 expression plasmid, and cells (1.2 × 10^5^) were seeded in 12-well plates. After cell attachment, cultured media was completely removed, cells were fixed and permeabilized with 100% methanol for 7 min, and blocked with 3% CAS-Blocking solution (Life Technologies, Carlsbad, CA, USA, Cat. 008120) for 15 min at RT. Cells were subsequently treated with rH5 mAb (1:100 dilution) for 1 h at RT and washed with PBS for three times. Alexa 488-conjugated anti-rabbit IgG (Invitrogen, Carlsbad, CA, USA, 1:500 dilution) was added and incubated for 1 h at RT. After washing, 4′,6-diamidino-2-phenylindole dihydrochloride (DAPI, 2 μg/mL) was used for nucleus staining. Stained cells were mounted on slides, covered with cover glass, and the fluorescence was detected by confocal microscope (Nikon, Tokyo, Japan) at 20× and 60× resolution.

### 2.18. Immunohistochemistry (IHC) Staining

IHC staining was performed using slides containing human lung tissues prepared at Severance Hospital-Gangnam, Seoul, Korea. Slides were deparaffinized as follows: Tissues were treated in xylene three times for 5 min each, 100% ethanol two times for 2 min each, 95% ethanol for 2 min, 90% ethanol for 2 min, 70% ethanol for 2 min, distilled water for 2 min, and 1× PBS for 5 min. The slides were then treated with 0.3% hydrogen peroxide for 10 min and rinsed with 1× PBS for 5 min. Next, they were microwaved in 0.1 M citrate buffer (pH 6.0) for 3.5 min, cooled down for 10 Sec, and boiled again for 10 Sec for antigen retrieval. These steps were repeated for 10 times and the slides were kept at RT for 20 min until the temperature dropped. After rinsing the slides with fleshly prepared 1× PBS three times for 5 min each, liquid barrier around the tissues were drawn using DAKO pen (DAKO). PBS with 2% normal goat serum (Jaximmuno) and 2% BSA (Sigma, Cat. A9647-100G) was added to each slide for 30 min at 4 °C for blocking. After removing all blocking solution, slides were incubated with primary antibody (rH5 mAb) within the boundary of liquid barrier and incubated overnight at 4 °C. Next day, the slides were rinsed with 1× PBS three times for 5 min each and the tissues were incubated with labeled polymer-HRP anti-mouse/rabbit antibody (DAKO) for 1 h at 4 °C. Tissues were washed with 1× PBS again, and treated with a mixture of 1 mL DAB+ substrate buffer and 20 µL DAB (3,3’-diaminobenzidine) chromogen for color fixation for 1 min under dark condition. Subsequently, counterstaining was performed by the treatment with Meyer’s hematoxylin (Sigma-Aldrich, St. Louis, MO, USA) for 1 min and rinse with running tap water. Tissues were dehydrated as follows: 70% ethanol (v/v) for 2 min, 90% ethanol for 2 min, 95% ethanol for 2 min, 100% ethanol twice for 2 min each, and xylene for three times for 5 min each. Drops of SUB-X mounting medium (Electron Microscopy Sciences, Hatfield, PA, USA) was added on tissues to place cover glass over them. Slides were scanned with Pannoramic MIDI (3D HISTECH).

### 2.19. Cell Viability Assay

HEK293T cells (4 × 10^4^) transfected with empty vector (EV) or DX2 mutant expression vector were seeded in 96-well plates and cultured for 24 h. MTT (3-(4,5-dimethylthiazol-2-yl)-2,5-diphenyl tetrazolium bromide, 5 mg/mL, Amresco) was added and incubated for 1 h at 37 °C. After discarding the media, 100 μL dimethylsulfoxide (DMSO, Duchefa) was added to each well for the solubilization of precipitated formazan. Absorbance at 560 nm was measured using a microplate reader. The experiments were repeated three times independently.

## 3. Results

### 3.1. Rabbit Immunization and Generation of Monoclonal Fab

**Figure 1:**
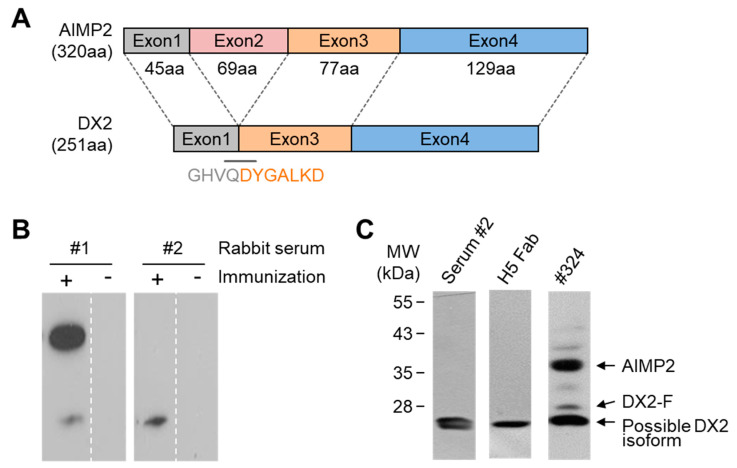
Rabbit immunization and the identification of H5 Fab. (**A**) Schematic drawing of the exon arrangement of AIMP2 (aminoacyl-tRNA synthetase-interacting multifunctional protein 2) (upper) and the exon 2-deleted splice variant AIMP2-DX2 (DX2, lower). The number of amino acids of each exon and the DX2-unique exon 1/3 junction sequence (GHVQDYGALKD) used for the immunization are indicated. (**B**) Immunoblotting was performed using pre- and post-immunized sera (- and +, respectively) isolated from each of the two rabbits to detect DX2 from H460 cell lysate. (**C**) H5 Fab, the polyclonal serum from rabbit #2, and clone #324 were used for immunoblotting of H460 cell lysate. Clone #324 recognizes both AIMP2 and full-size DX2 (DX2-F, ~28 kDa) along with other unidentified proteins. The small ~25 kDa protein recognized by both H5 Fab and clone #324 was indicated as a possible DX2 isoform. Full blots for Figure 1B,C are provided in Figure S5.

To generate an antibody specific to DX2, two rabbits were immunized with a short KLH-conjugated peptide, GHVQDYGALKD-Cys, spanning the exon 1/3 junction region of DX2 as the antigen (Figure 1A). After immunization, we performed immunoblotting with the sera of the rabbits using H460 lung cancer cell lysates expressing a high level of DX2. The result revealed that one of the rabbits (rabbit #2) produced a serum that showed a strong band near the estimated molecular weight (MW) of DX2 (27.8 kDa) whereas the serum from the other rabbit exhibited two bands whose sizes were similar to those of AIMP2 and DX2 (Figure 1B). We compared the #2 rabbit serum with clone #324 (Neomics) and found that the protein recognized by #2 rabbit serum corresponded with the lowest band detected by clone #324, and was slightly smaller than the expected MW of DX2 (Figure 1C). Although the protein recognized by the rabbit serum did not exactly match DX2, we reasoned that it could be an isoform of DX2. Therefore, we constructed an antibody library to generate a mAb using the total mRNA from the spleen of #2 rabbit. 

Total mRNA was extracted from the spleen of #2 rabbit and used for the synthesis of cDNA from which the antibody variable regions were amplified by PCR (Tables S1 and S2 and Figure S1). Fabs were generated by two steps of overlap-extension PCR (Table S3) and ligated into pComb3X vector, and the ligation mixture was transformed into *E. coli* ER2537 competent cells. Finally, a phage library of a total of 7.2 × 10^7^ clones was generated and used for panning against the BSA-conjugated DX2 peptide. Three rounds of panning selection were conducted with gradual increase in the number of washes, followed by ELISA (enzyme-linked immunosorbent assay) screening. H5 Fab was found to be the only clone that specifically recognized the same protein detected by the serum of rabbit #2 in immunoblot (Figure 1C). 

### 3.2. Validation of H5 Antibody

**Figure 2:**
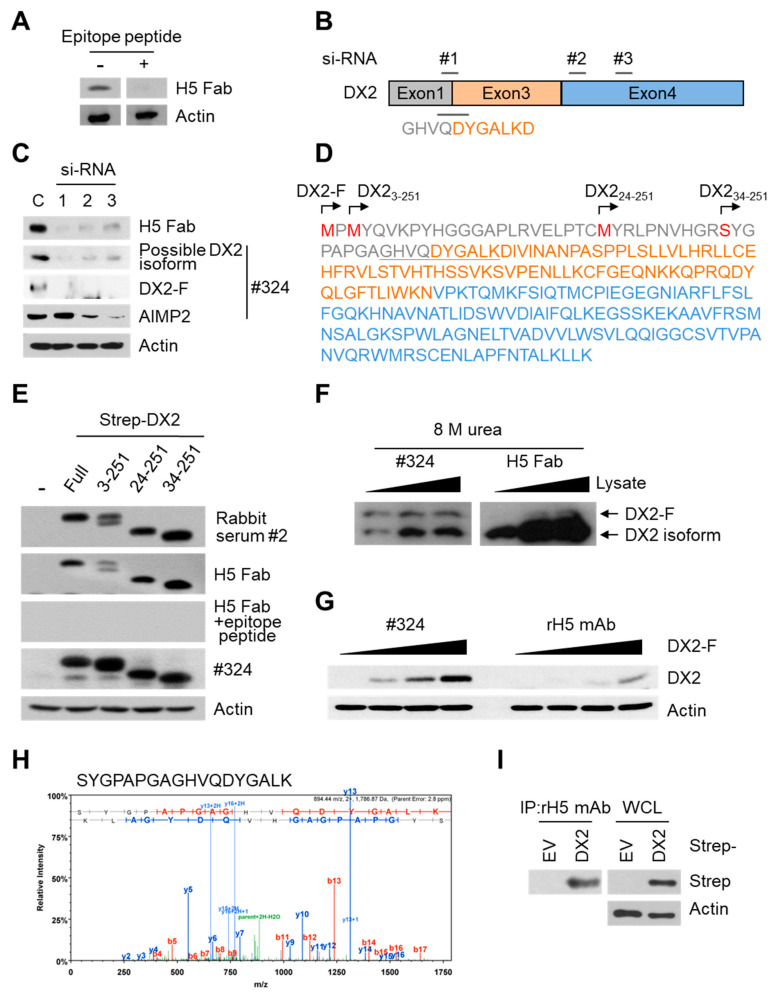
Validation of H5. (**A**) The masking effect of a DX2 epitope peptide, GAGHVQDYGALK, against the immunoblot band recognition by H5 Fab. The peptide was incubated with *Escherichia coli* extract containing H5 Fab during the incubation step of immunoblotting with H460 cell lysate. Actin was used as a loading control. The experiment was performed in duplicate. (**B**) The sites of siRNA hybridization in the DX2 mRNA. #1, #2, and #3 indicate three different si-RNAs against DX2 mRNA. (**C**) Levels of the possible DX2 isoform, DX2-F, and AIMP2 were detected by H5 Fab and clone #324 in the si-DX2-transfected H460 cell lysates. Three different si-DX2 sequences (#1~#3) were used to knock down the expression of DX2 in H460 cells. The experiment was performed in duplicate. C, negative control. (**D**) Amino acid sequences of the DX2 isoforms transfected into H460 cells. The initiating amino acid (Met) in each of the DX2 isoforms was indicated in red. Grey, orange, and blue colors represent exon 1, exon 3, and exon 4 sequences of DX2, respectively. (**E**) Strep-tagged DX2 isoforms were transfected into H460 cells and detected by #2 rabbit serum, H5 Fab, and clone #324 by immunoblotting. Masking effect of the DX2 epitope peptide was also investigated by mixing the epitope peptide with H5 Fab. The experiment was performed in duplicate. (**F**) H460 cell lysates prepared in 8 M urea were subjected to SDS-PAGE and immunoblotting using clone #324 and H5 Fab. The experiment was performed in duplicate. (**G**) HEK293T cells in 6-well plates were transfected with 0, 1, 2, and 3 μg of tag-free DX2-F and subjected to immunoblotting with clone #324 and H5 Fab. The experiment was performed in duplicate. (**H**) Identification of unique peptide sequence of DX2 from the proteins immunoprecipitated by rH5 antibody. Strep-DX2-expressing HEK293T cell lysates were immunoprecipitated with rH5 antibody and subjected to mass spectrometry analysis (See Figure S3). Sequence of the identified exon 1/3 junction peptide is shown on top. (**I**) An aliquot of the same sample used for mass spectrometry was also subjected to SDS-PAGE and immunoblotting using antibody against Strep-tag for the confirmation of immunoprecipitation. Full blots for Figure 2A,C,E–G,I are provided in Figure S6.

To confirm that H5 Fab recognizes the immunogen peptide sequence, an exon 1/3 junction peptide, GAGHVQDYGALK, was mixed with the E. coli extract containing H5 Fab for immunoblot analysis of H460 cell lysate. The competing peptide has two additional amino acids at the N-terminus and lacks the C-terminal aspartate compared with the immunogen peptide, and we reasoned that a true exon 1/3 junction-specific antibody would recognize both sequences. The signal from H5 Fab disappeared by adding the peptide, proving the specific recognition of the exon 1/3 junction sequence by H5 Fab (Figure 2A). Next, three different siRNAs were transfected into H460 cells to knockdown DX2 (Figure 2B) and immunoblotting using H5 Fab and clone #324 was performed. The results revealed that the signal from H5 Fab significantly declined after siRNA transfection (Figure 2B,C), similarly to those of the full-length DX2 (DX-F, a weak ~28 kDa band) and the lower MW band (a possible DX2 isoform) detected by clone #324. Especially, these signals almost disappeared by transfecting si-DX2 #1 specific to the exon 1/3 junction, implying that the protein recognized by H5 Fab was derived from DX2. 

The band recognized by H5 Fab appeared to be slightly smaller than DX2-F (27.8 kDa). To test whether this band is a smaller isoform of DX2 and whether H5 Fab can detect full-length DX2, DX2-F, DX2_3-251_, DX2_24-251_, and DX2_34-251_ genes with N-terminal Strep-tag were transfected to H460 cells and immunoblotted with H5 Fab. The three DX2 isoforms, DX2_3-251_, DX2_24-251_, and DX2_34-251_ (Figure 2D), were prepared because Met2 and Met23 could act as an alternative initiation site and the peptide bond between the amino acid positions 33 and 34 (Arg-Ser) was predicted to be a potential enzymatic cleavage site based on the PeptideCutter analysis [21]. Interestingly, all the DX2 isotypes as well as DX2-F were detected by #2 rabbit serum and H5 Fab as well as by clone #324 (Figure 2E). When the epitope peptide was mixed with H5 Fab, all the signals from the overexpressed DX2-F and DX2 isoforms disappeared as expected (Figure 2E, third panel). These data suggested that H5 Fab can recognize DX2 and its isoforms as long as they harbor the exon 1/3 junction sequence. Therefore, we designate the protein recognized by H5 Fab as an endogenous DX2 isoform hereafter. 

The overexpression of Strep-DX2-F and its detection by H5 Fab (Figure 2E) raised a question as to why endogenous DX2-F was not detected with H5 Fab even though it harbors the exon 1/3 junction. To solve this question, immunoblotting was performed using 8 M urea sample buffer (Figure 2F). It showed that H5 Fab could recognize an additional band similar in size to endogenous DX2-F under the strong denaturing condition of 8 M urea sample buffer, albeit with a weak signal (Figure 2F). It also implied that the exon 1/3 junction region of DX2-F may not be fully accessible by H5 Fab even when it is denatured in SDS sample buffer (Figure 2E). While SDS is believed to almost fully denature protein conformation, reports have been made of rare cases where noncovalent inter- or intramolecular interactions are retained upon SDS treatment [22,23]. An intriguing point was that H5 Fab recognizes Strep-tagged DX2-F more effectively than tag-free endogenous DX2-F (Figure 2E,F). It seems that the N-terminal tag is critical for the exposure of the epitope sequence, possibly by depriving the structural flexibility from the N-terminus of DX2-F not to mask the exon 1/3 junction. Nonetheless, it is clear that the DX2 isoform is predominant over DX2-F based on the immunoblot results of H5 and clone #324 (Figure 1C and Figure 2F). It is plausible that there are specific circumstances or underlying mechanisms by which the endogenous DX2 isoform is generated. Taken together, these results strongly suggested that the major form of DX2 recognized by H5 Fab is the DX2 isoform containing exon 1/3 junction; however, the exact sequence of the isoform or the mechanism of its generation could not be elucidated from the data.

After validating H5 Fab as an anti-DX2 antibody, we converted it to rabbit (rH5) and human–rabbit chimeric (hH5) IgGs. We compared the specificity of rH5 mAb to purified DX2_34-251_ and AIMP2_34-320_ with that of clone #324. While clone #324 detected both DX2_34-251_ and AIMP2_34-320_, rH5 mAb did not recognize AIMP2_34-320_ in ELISA (Figure S2), proving that H5 mAb is specific to the exon 1/3 junction region sequence unique to DX2. We also performed immunoblotting using rH5 mAb and clone #324 at 4 μg/mL, since both antibodies showed similar ELISA signal levels to DX2_34-251_ at that concentration. We transfected HEK293T cells with varying levels of tag-free DX2-F DNA and observed that rH5 mAb could detect tag-free DX2-F when the expression level of tag-free DX2-F was high (Figure 2G). It showed that the level of DX2-F is another critical factor for rH5 mAb to recognize its target protein. 

Since H5 mAbs best recognize DX2-F at a high level and/or with an N-terminal tag, we transfected Strep-tagged DX2-F into HEK293T cells and performed immunoprecipitation with rH5 mAb for LC-MS analysis (Figure S3). As expected, the mass spectrometry analysis identified the specific exon 1/3 junction peptide of DX2, SYGPAPGAGHVQDYGALK (Figure 2H), confirming the ability of H5 mAb to recognize the sequence. An aliquot of the sample used for the LC-MS analysis was also subjected to immunoblotting; Strep-tagged DX2-F was successfully detected by anti-Strep tag antibody (Figure 2I). 

### 3.3. Characterization of DX2 Isoform Detected by rH5 mAb

**Figure 3:**
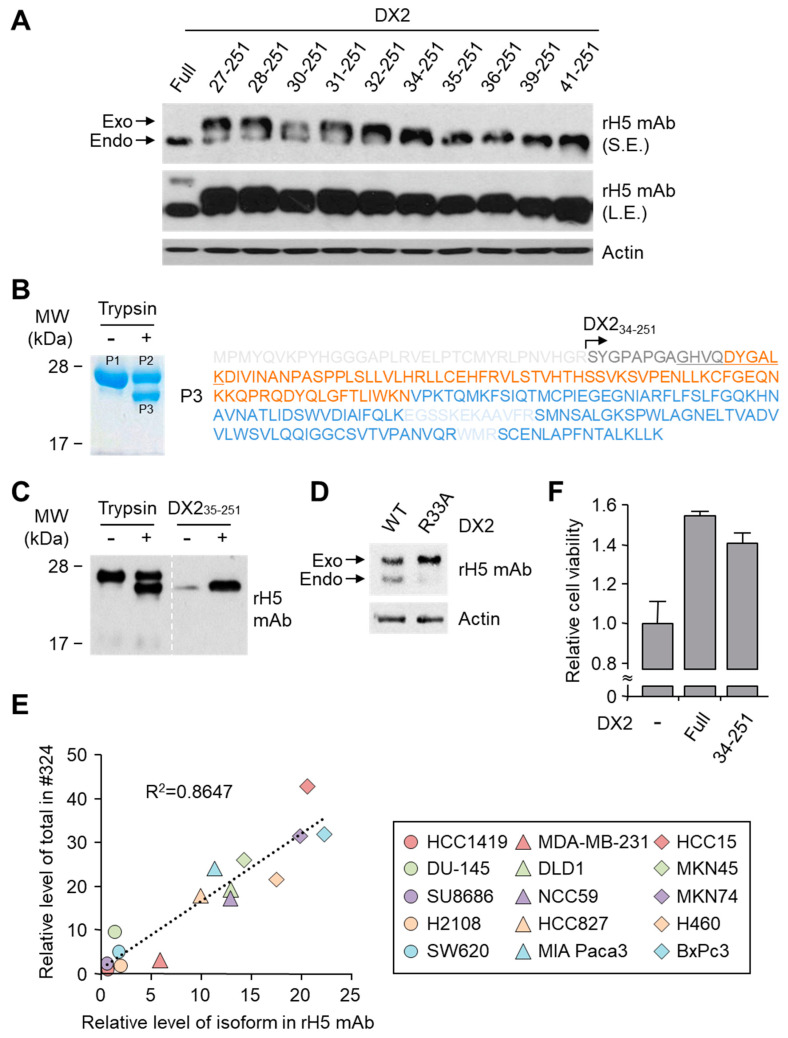
Identification of DX2 isoform recognized by H5 mAb. (**A**) Two μg each of DNA for DX2-F and predicted isoforms (See Supplemental Table S4) was transfected into HEK293T cells seeded in 6-well plates and the cell lysates were subjected to SDS-PAGE and immunoblotting using rH5 antibody. Transfected proteins and the endogenous DX2 isoforms are indicated as exo and endo, respectively. Actin was used as a loading control. S.E., short exposure; L.E., long exposure. (**B**) Limited proteolysis of the purified DX2-F protein. In vitro-purified DX2-F protein (P1) was treated with trypsin and electrophoresed by SDS-PAGE. Trypsin-treated proteins (P2 and P3) were visualized by Coomassie staining (left) and analyzed by LC-MS analysis. Identified sequences of DX2 are presented here (P3) and in Supplemental Figure S4A (P1 and P2). Light grey letters indicate undetected sequences. The color scheme is identical to Figure 2D. (**C**) The band sizes of DX2-F treated with trypsin were compared with those of endogenous DX2 isoform and exogenous DX2_35-251_ expressed in HEK293T by immunoblotting using rH5 antibody. (**D**) Generation of the DX2 isoform from the ectopically introduced DX2 wild type (WT) and the R33A mutant in CHO-K1 cell lysates. (**E**) Linear correlation between the levels of total DX2 and the DX2 isoform, quantified by immunoblotting with clone #324 and rH5 antibody, respectively, in various cancer cell lines. (**F**) Proliferation of HEK293T cells expressing DX2-F and DX2_34-251_ was investigated by MTT assay. The proliferation data is presented as mean and standard deviation (*n* = 3). Full blots/gel for Figure 3A–D are provided in Figure S7. All experiments were performed in duplicate (**A**–**E**) or triplicate (**F**).

H5 antibody can recognize both DX2-F as well as DX2 isoforms; however, the latter seems to be the dominant form existing in cancer cells as revealed by the immunoblotting with clone #324 antibody (Figure 1C). The DX2 isoform appears to be smaller than DX2_3-251_ or DX2_23-251_ which can be generated from different open reading frames (Figure 2E); therefore, we searched for other possible isoforms produced by proteolytic digestion using PeptideCutter to identify the dominant DX2 isoform (Table S4) [21]. We cloned all the possible DX2 isoforms by deleting corresponding N-terminal sequences and incorporating additional ATG codon in front of the open reading frame to initiate translation. We overexpressed each DX2 isoform in HEK293T cells and compared their sizes with that of the endogenous DX2 isoform. As expected, rH5 mAb detected all the introduced DX2 isoforms better than DX2-F and the latter was only weakly recognized only under the condition of a long exposure time (Figure 3A). According to the immunoblotting result, the size of the expressed DX2_35-251_ isoform matched that of the endogenous isoform (Figure 3A, first panel). Considering that the transfected DX2_35-251_ isoform harbors additional Met for ectopic expression, the actual size of the endogenous form may correspond to that of N-terminal 33 aa-deleted DX2 (DX2_34-251_) if the N-terminal Met of DX2_35-251_ was not excised post-translationally [24]. 

To test this hypothesis, we treated purified DX2-F protein with trypsin, a well-known protease, and the cleaved proteins were subjected to LC-MS analysis to identify the cleaved sites. Sequence of undigested DX2-F protein (P1 and P2) was fully identified (Figure S4A) but the sequence of the cleaved protein (P3) started from Ser34 (Figure 3B), showing that N-terminal 33 aa were cleaved by trypsin. Moreover, the size of DX2 cleaved by trypsin seemed to closely match that of the endogenous isoform as well as of transfected DX2_35-251_ as revealed by immunoblotting using rH5 mAb (Figure 3C), suggesting that 33 aa-cleaved DX2 (DX2_34-251_) could be the endogenous DX2 isoform detected by rH5 mAb. To further investigate whether DX2_34-251_ is the dominant isoform, we cloned the cleavage-defective DX2 R33A mutant, transfected it into cells, and compared the levels of isoform generation. While the cells transfected with DX2-F wild type (WT) showed clear band of DX2 isoform, the cells with the mutant form revealed only a faint band (Figure 3D), implying that the dominant endogenous isoform could be DX2_34-251_. 

We further compared the levels of DX2-F and the isoform detected by clone #324 and rH5 mAb in various cancer cell lines. A well-fitted positive correlation was determined among the tested cell lines (Figure 3E and Figure S4B), indicating that the level of the isoform detected by rH5 mAb is indicative of the level of total DX2 detected by clone #324. Finally we investigated whether the identified DX2 isoform DX2_34-251_ exhibited oncogenic function similar to DX2-F, and observed that the isoform efficiently enhanced cell proliferation similarly to DX2-F (Figure 3F). All these results suggested that DX2 isoform detected by rH5 mAb could be a viable surrogate for studying the expression as well as function of DX2-F.

### 3.4. Binding Affinity and Specificity of H5 mAb

**Figure 4:**
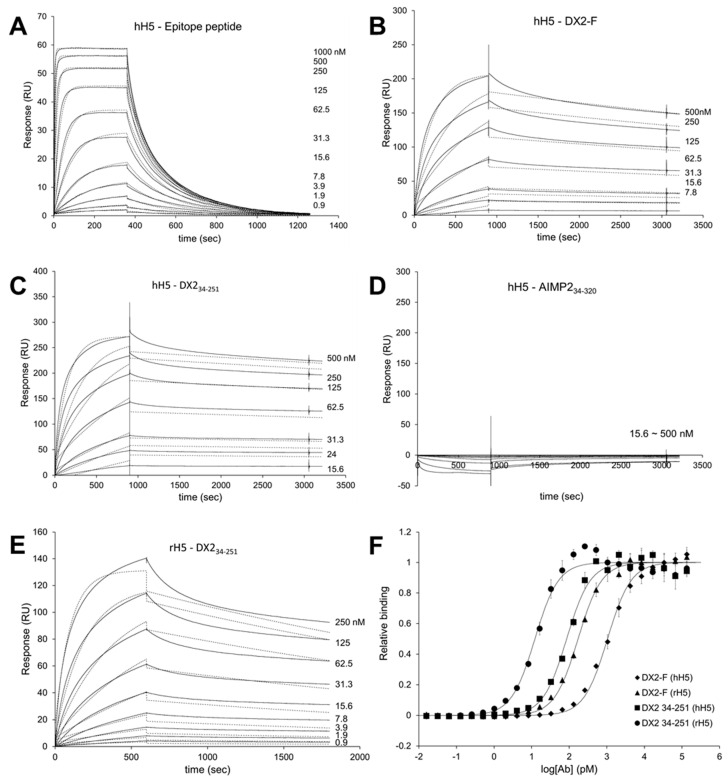
Determination of binding affinity of H5 antibody to DX2. (**A**–**E**) Binding affinities of H5 antibody to the epitope peptide, DX2-F, and DX2 isoform were determined by SPR assay. Sensorgrams of the binding of hH5 antibody to the epitope peptide (**A**), DX2-F (**B**), DX_234-251_ (**C**), and AIMP2_34-320_ (**D**), and those of rH5 antibody to DX2_34-251_ (**E**) are shown. Solid lines: actual sensorgram data, dotted lines: sensorgram data fitted to 1:1 Langmuir binding model. (**F**) Binding affinities of hH5 and rH5 antibodies to DX2-F and DX2_34-251_ proteins were measured by ELISA. The binding parameters analyzed by SPR and ELISA are summarized in Table 1 and Table 2, respectively. All experiments were performed in duplicate.

To investigate the binding affinity of H5 mAb to the epitope peptide, GAGHVQDYGALK, and purified proteins, including DX2-F, DX2_34-251_, and AIMP2_34-320_, SPR analysis was performed. The equilibrium affinity constant (*K*_D_) value of hH5 mAb to the epitope peptide, DX2-F and DX2_34-251_ were measured at 22.5 nM, 9.42 nM, and 3.23 nM, respectively (Table 1 and Figure 4A–C). hH5 mAb did not show any interaction with AIMP2_34-320_ (Table 1 and Figure 4D). rH5 and hH5 mAbs showed similar binding affinity to DX2_34-251_ based on *K*_D_ values (Table 1 and Figure 4E). We also analyzed the binding affinity of hH5 and rH5 mAbs to DX2-F and DX2_34-251_ by ELISA. Both antibodies exhibited about 3.3- to 13.9-fold higher binding affinity to DX2_34-251_ than to DX2-F, and rH5 mAb exhibited stronger binding to antigens than hH5 (Table 2 and Figure 4F). 

It is noteworthy that H5 mAb shows nanomolar affinity to DX2_34-251_ as well as to DX2-F in vitro, although the affinity for DX2_34-251_ is higher than for DX2-F. It implies that the relatively low detection level of endogenous DX2-F in cancer cells by H5 antibodies would be due to the unique intracellular environment of cancer cells facilitating the production of DX2_34-251_ from DX2-F or giving more structural flexibility to N-terminal DX2-F to mask the exon 1/3 junction sequence. 

### 3.5. Application of H5 mAb to Immunostaining

**Figure 5:**
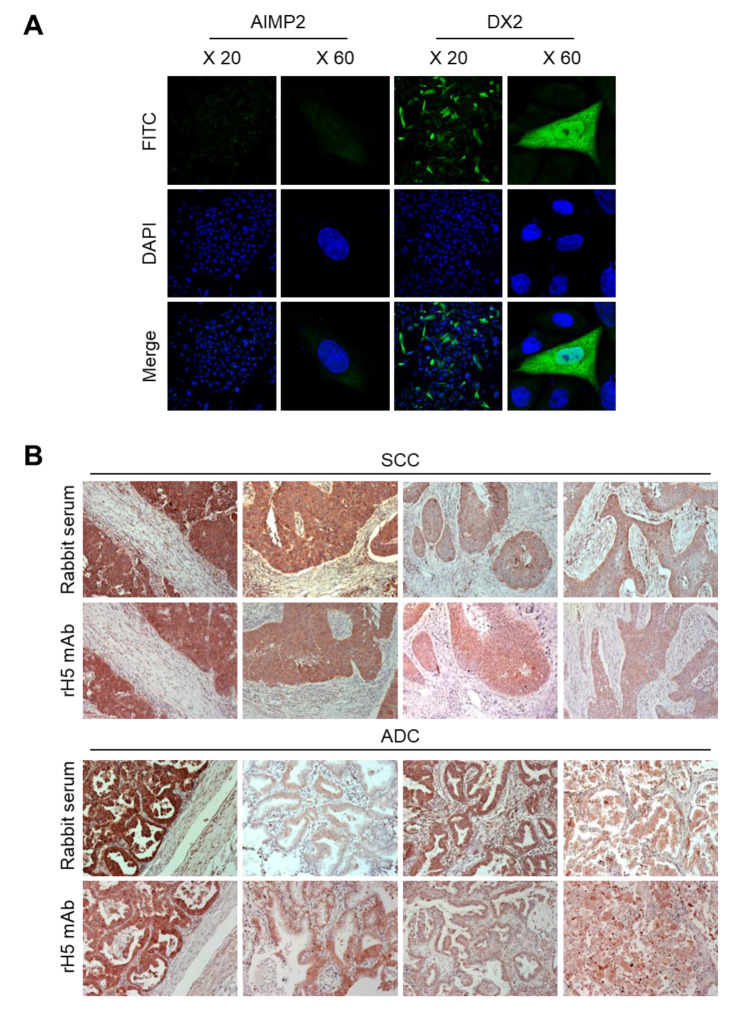
Immunostaining with H5 mAb. (**A**) CHO-K1 cells expressing Strep-AIMP2 or -DX2 were subjected to immunofluorescence staining with rH5 antibody. DX2 and nucleus were visualized by FITC and DAPI, respectively. The images are presented at 20× and 60× magnifications. (**B**) Levels of DX2 in tissues from human squamous cell carcinoma (SCC) and adenocarcinoma (ADC) of the lung were determined by immunohistochemistry staining using #2 rabbit serum and rH5 antibody. The images are presented at 200× magnification. All experiments were performed in duplicate.

Since H5 mAb shows high affinity and specificity to DX2 isoform in cellular condition, we performed immunofluorescence (IF) staining to investigate whether H5 mAb is applicable for IF. Strep-DX2 or -AIMP2 was overexpressed in CHO-K1 cells and stained with rH5 mAb. While we did not observe any clear signal from AIMP2-expressing cells (Figure 5A, left), DX2-expressing cells showed clear green fluorescence signal after rH5 mAb staining (Figure 5A, right), suggesting that rH5 mAb is applicable to IF staining. We also applied rH5 mAb to IHC staining using human lung cancer tissues. It had previously been shown that AIMP2-DX2 is strongly expressed in SCC (squamous cell carcinoma) and ADC (adenocarcinoma) tissues of the lung, with a significant correlation with cancer stage [10]. The IHC staining pattern using rH5 mAb was very similar to that of the rabbit polyclonal serum, with strong staining intensity on the tumor tissues (Figure 5B). In contrast, normal lung tissues were not stained or only weakly stained by the same antibody (Figure S8), implying cancer-specific expression of DX2 isoform in both SCC and ADC. The data suggested the possibility that H5 mAb can be applied as a diagnostic tool to screen for cancer patients with high expression of DX2. 

## 4. Discussion

Previous studies have shown that DX2 compromises the tumor suppressive activity of AIMP2 and promotes tumor progression [10]. It is already known that high expression of DX2 in patients with cancer is significantly correlated with poor prognosis and chemoresistance in lung cancer and ovarian cancer, respectively [7,25], and knockdown or suppression of DX2 induced tumor regression [10,12], suggesting that DX2 could be a potential therapeutic target and diagnostic marker. Over the past years, multiple efforts to create an antibody against DX2 have proven unsuccessful despite its potential applicability as a diagnostic tool, due to the poor stability and immunogenicity of DX2 protein. Various forms of purified DX2 proteins with different tags and modifications to improve its solubility and stability, as well as DX2 exon 1/3 junction sequence peptides of different lengths were used as immunogens for rabbits and mice as well as for antibody library panning; however, no antibody with specificity toward DX2 could be identified. 

In this study, we immunized rabbits with a short peptide, GHVQDYGALKD, specific to exon 1/3 junction of DX2. Remarkably, one of the two immunized rabbits yielded a polyserum that showed a strong and specific immunoblotting band roughly corresponding to the molecular weight of DX2. We generated a phage Fab library from the splenic cDNA of the rabbit, from which H5 mAb was isolated. The size of the Fab library prepared for this study was estimated to be 7.2 × 10^7^ clones as deduced from the number of *E. coli* transformants. While this number is somewhat low for an antibody library, the immunized antibody repertoire was already enriched with target binders and prominent binding clones could be isolated, even with VH-VL shuffling that might considerably dilute native VH-VL pairings in the original immune repertoire. The library was panned against the exon 1/3 junction sequence peptide conjugated to BSA, and multiple peptide binders were identified by ELISA screening of the panning output clones. Of these, only one clone could detect the endogenous DX2 isoform protein in H460 cell lysate in immunoblot assay, possibly due to the difference between the conformations of the BSA-conjugated peptide and the corresponding sequence in SDS-denatured protein, or because some of the clones recognized a part of the linker structure that conjugated the peptide to BSA. The conformational dependence of the antibody binding to the exon 1/3 junction sequence may also be the reason for the dependence of H5 binding to DX2 on the N-terminal sequence of the antigen, possibly via flexibility-derived conformational differences.

The clone H5 could be utilized in immunoblotting, immunoprecipitation, ELISA, IF, and IHC experiments, showing its applicability to research purposes as well as to clinical diagnosis. While clone #324 recognizes DX2 as well as AIMP2, H5 monoclonal antibody specifically detects DX2. Although the epitope of clone #324 had not been identified yet, it is in the exon 3-exon 4 region since clone #324 was produced by immunizing a mouse with AIMP2_84-225_ which includes parts of exons 2 and 4 and the entire exon 3 [6], and can detect AIMP2, DX2-F, and DX2 isoforms altogether. Compared with clone #324, H5 showed much weaker recognition of DX2-F, which suggested that N-terminal region of DX2-F interacts with the exon 1/3 junction and thereby interferes with H5′s access to the region. The exon 1-exon 2 region of AIMP2 includes a leucine-zipper motif [26], but following splicing of the exon 2, it is plausible that the remaining exon 1 loses structural stability. Actually DX2-F was difficult to be crystalized (unpublished data) and an insertion of N-terminal tag significantly enhanced the detectability of DX2-F by H5, corroborating the importance of structural stability of DX2 N-terminus for its recognition by H5. We searched the possible identity of the endogenous DX2 isoform by analyzing the N-terminal digestion by proteases, but not C-terminal digestion, since C-terminal exon 3-exon 4 consists of a stable GST (glutathione S-transferase) domain [26]. It is likely that the N-terminus of DX2-F is unstable in intracellular environment and proteolytically cleaved to the shorter isoform, which may explain the low level of detection by #324 and the marginal recognition by H5 of DX2-F in H460 cell lysate by immunoblotting. We focused on identifying the N-terminal sequence of the DX2 isoform. Based on the results from mutation analysis, limited digestion, and mass spectrometry, we propose the isoform detected by H5 mAb to be DX2_34-251_, which could be generated by cleavage between Arg33 and Ser34 by proteases, such as trypsin (Figure 3). It is unclear how the DX2-F is actually processed by proteolytic enzymes to generate DX2_34-251_ within cancer cells. Several reports have suggested that cancer cells express proteolytic enzymes, including trypsin [27,28]; however, more studies are required to elucidate the underlying mechanism of DX2 isoform generation and its relationship to protease enzymes such as trypsin. 

While DX2-F is detected much more weakly by H5 than by clone #324, DX2_34-251_ is effectively recognized by H5 and functions like DX2-F by enhancing cell proliferation (Figure 3F). The level of DX2_34-251_ is strongly correlated with the total level of DX2 (DX2-F and its isoform combined). Taken together, these results suggested that DX2_34-251_ could be used as a surrogate marker for DX2-F and sensitively detected by H5. The development of H5 mAb expanded the limitation of clone #324, which is only applicable to immunoblotting because the antibody detects both AIMP2 and DX2. In the era of precision medicine for cancer therapy, it is expected that the H5 antibody has the potential to be a useful research tool as well as a diagnostic agent for several solid tumors associated with DX2 overexpression, in IHC, cytology staining, or point-of-care testing kits. H5 mAb is currently being tested in multiple studies for the diagnosis of cancers including hematological and colorectal cancers, with increased numbers of patients to ensure proper clinical validation of DX2 and H5 (manuscripts submitted or in preparation).

## 5. Conclusions

AIMP2-DX2 is selectively overexpressed in many cancers, and its expression level is correlated with the clinical outcome of the treatment, making it an ideal cancer diagnostic marker and a potential cancer treatment target. However, the generation of AIMP2-DX2 specific antibodies has been hampered by the poor stability and low immunogenicity of the splice variant, which in turn significantly delayed the biological characterization and the development of diagnosis for this important cancer marker. The novel monoclonal antibody H5 specifically recognizes AIMP2-DX2 with a preference for the isoform(s) with N-terminal truncation; our study suggests that the predominant isoform of AIMP2-DX2 is likely to have the first 33 amino acids cleaved by one or more intracellular proteases, the identity and the significance of which requiring further research efforts. Nonetheless, the AIMP2-DX2 isoform detected by H5 mAb is a viable surrogate for the full-length AIMP2-DX2 (DX2-F), and H5 mAb is expected to be a useful capture and detection agent for research and diagnosis of AIMP2-DX2-expressing cancers.

## 6. Patents

The work reported in this manuscript has been applied for patents in the United States, the European Union, China, Japan, and Korea.

## Figures and Tables

**Table 1:** Kinetic parameters of H5 binding to its targets measured by Surface Plasmon Resonance (SPR). N.B., no binding.

Antibody	Ligands	*k*_a_ (M^−1^ s^−1^)	*k*_d_ (s^−1^)	*K*_D_ (nM)
hH5	Epitope peptide	3.56 × 10^5^	8.03 × 10^−3^	22.5
	DX2-F	9.06 × 10^3^	8.53 × 10^−5^	9.42
	DX2_34-251_	1.34 × 10^4^	4.35 × 10^−5^	3.23
	AIMP2_34-320_	N.B.	N.B.	N.B.
rH5	DX2_34-251_	3.98 × 10^4^	2.57 × 10^−4^	6.46

**Table 2:** Affinity of H5 to DX2, measured by ELISA. BC_50_, half maximal binding concentration.

Antibody	Ligands	BC_50_ (pM)
hH5	DX2-F	276.8
	DX2_34-251_	83.9
rH5	DX2-F	172.3
	AIMP2_34-320_	12.4

## Supplementary Materials

The following are available online at https://www.mdpi.com/2218-273X/10/6/820/s1, Table S1: Primers designed for the amplification of rabbit variable region genes, Table S2: Primers used for RT-PCR, Table S3: Primers designed for overlap extension PCR to join fragments into Fab, Table S4: Cleavage sites in DX2 between amino acid positions 25–41, predicted by PeptideCutter program, Figure S1: Cloning process of Fab fragment generation, Figure S2: ELISA of clone #324 and rH5 antibodies, Figure S3: Strategy for the identification of exon 1/3 junction peptide of DX2 from the immunoprecipitated protein using rH5 antibody and subsequent mass spectrometry analysis, Figure S4: Identification of DX2 isoform detected by H5, Figure S5: Full images of Western blot membranes for Figure 1, Figure S6: Full images of Western blot membranes for Figure 2, Figure S7: Full images of Western blot membranes for Figure 3, Figure S8: Immunostaining of normal lung tissues with H5 mAb.

## References

[1] Agarwal A., Ressler D., Snyder G. (2015). The current and future state of companion diagnostics. Pharm. Pers. Med..

[2] Korpanty G.J., Graham D.M., Vincent M.D., Leighl N.B. (2014). Biomarkers That Currently Affect Clinical Practice in Lung Cancer: EGFR, ALK, MET, ROS-1, and KRAS. Front. Oncol..

[3] Kim S., You S., Hwang D. (2011). Aminoacyl-tRNA synthetases and tumorigenesis: More than housekeeping. Nat. Rev. Cancer.

[4] Kim M.J., Park B.J., Kang Y.S., Kim H.J., Park J.H., Kang J.W., Lee S.W., Han J.M., Lee H.W., Kim S. (2003). Downregulation of FUSE-binding protein and c-myc by tRNA synthetase cofactor p38 is required for lung cell differentiation. Nat. Genet..

[5] Kim D.G., Lee J.Y., Lee J.H., Cho H.Y., Kang B.S., Jang S.Y., Kim M.H., Guo M., Han J.M., Kim S.J. (2016). Oncogenic Mutation of AIMP2/p38 Inhibits Its Tumor-Suppressive Interaction with Smurf2. Cancer Res..

[6] Choi J.W., Kim D.G., Park M.C., Um J.Y., Han J.M., Park S.G., Choi E.C., Kim S. (2009). AIMP2 promotes TNFalpha-dependent apoptosis via ubiquitin-mediated degradation of TRAF2. J. Cell Sci..

[7] Han J.M., Park B.J., Park S.G., Oh Y.S., Choi S.J., Lee S.W., Hwang S.K., Chang S.H., Cho M.H., Kim S. (2008). AIMP2/p38, the scaffold for the multi-tRNA synthetase complex, responds to genotoxic stresses via p53. Proc. Natl. Acad. Sci. USA.

[8] Yum M.K., Kang J.S., Lee A.E., Jo Y.W., Seo J.Y., Kim H.A., Kim Y.Y., Seong J., Lee E.B., Kim J.H. (2016). AIMP2 Controls Intestinal Stem Cell Compartments and Tumorigenesis by Modulating Wnt/beta-Catenin Signaling. Cancer Res..

[9] Choi J.W., Lee J.W., Kim J.K., Jeon H.K., Choi J.J., Kim D.G., Kim B.G., Nam D.H., Kim H.J., Yun S.H. (2012). Splicing variant of AIMP2 as an effective target against chemoresistant ovarian cancer. J. Mol. Cell Biol..

[10] Choi J.W., Kim D.G., Lee A.E., Kim H.R., Lee J.Y., Kwon N.H., Shin Y.K., Hwang S.K., Chang S.H., Cho M.H. (2011). Cancer-associated splicing variant of tumor suppressor AIMP2/p38: Pathological implication in tumorigenesis. PLoS Genet.

[11] Oh A.Y., Jung Y.S., Kim J., Lee J.H., Cho J.H., Chun H.Y., Park S., Park H., Lim S., Ha N.C. (2016). Inhibiting DX2-p14/ARF Interaction Exerts Antitumor Effects in Lung Cancer and Delays Tumor Progression. Cancer Res..

[12] Lee H.S., Kim D.G., Oh Y.S., Kwon N.H., Lee J.Y., Kim D., Park S.H., Song J.H., Lee S., Han J.M. (2013). Chemical suppression of an oncogenic splicing variant of AIMP2 induces tumour regression. Biochem. J..

[13] Shukla A., Das Bhowmik A., Hebbar M., Rajagopal K.V., Girisha K.M., Gupta N., Dalal A. (2018). Homozygosity for a nonsense variant in AIMP2 is associated with a progressive neurodevelopmental disorder with microcephaly, seizures, and spastic quadriparesis. J. Hum. Genet..

[14] Walker S.E., Lorsch J. (2013). RNA purification--precipitation methods. Methods Enzymol..

[15] Kay B.K., Kasanov J., Yamabhai M. (2001). Screening phage-displayed combinatorial peptide libraries. Methods.

[16] Yang H.Y., Kang K.J., Chung J.E., Shim H. (2009). Construction of a large synthetic human scFv library with six diversified CDRs and high functional diversity. Mol. Cells.

[17] Jha R., Cho H.Y., Mushtaq A.U., Lee K., Kim D.G., Kim S., Jeon Y.H. (2017). Purification and biophysical characterization of the AIMP2-DX2 protein. Protein Expr. Purif..

[18] Lee J.Y., Kim D.G., Kim B.G., Yang W.S., Hong J., Kang T., Oh Y.S., Kim K.R., Han B.W., Hwang B.J. (2014). Promiscuous methionyl-tRNA synthetase mediates adaptive mistranslation to protect cells against oxidative stress. J. Cell Sci..

[19] Keller A., Nesvizhskii A.I., Kolker E., Aebersold R. (2002). Empirical statistical model to estimate the accuracy of peptide identifications made by MS/MS and database search. Anal. Chem..

[20] Nesvizhskii A.I., Keller A., Kolker E., Aebersold R. (2003). A statistical model for identifying proteins by tandem mass spectrometry. Anal. Chem..

[21] Wilkins M.R., Gasteiger E., Bairoch A., Sanchez J.C., Williams K.L., Appel R.D., Hochstrasser D.F. (1999). Protein identification and analysis tools in the ExPASy server. Methods Mol. Biol..

[22] Zolotarjova N.I., Hollis G.F., Wynn R. (2001). Unusually stable and long-lived ligand-induced conformations of integrins. J. Biol. Chem..

[23] Neefjes J.J., Ploegh H.L. (1992). Inhibition of endosomal proteolytic activity by leupeptin blocks surface expression of MHC class II molecules and their conversion to SDS resistance alpha beta heterodimers in endosomes. EMBO J..

[24] Giglione C., Boularot A., Meinnel T. (2004). Protein N-terminal methionine excision. Cell. Mol. Life Sci..

[25] Choi J.W., Um J.Y., Kundu J.K., Surh Y.J., Kim S. (2009). Multidirectional tumor-suppressive activity of AIMP2/p38 and the enhanced susceptibility of AIMP2 heterozygous mice to carcinogenesis. Carcinogenesis.

[26] Guo M., Yang X.L., Schimmel P. (2010). New functions of aminoacyl-tRNA synthetases beyond translation. Nat. Rev. Mol. Cell Biol..

[27] Soreide K., Janssen E.A., Korner H., Baak J.P. (2006). Trypsin in colorectal cancer: Molecular biological mechanisms of proliferation, invasion, and metastasis. J. Pathol..

[28] Yamashita K., Mimori K., Inoue H., Mori M., Sidransky D. (2003). A tumor-suppressive role for trypsin in human cancer progression. Cancer Res..

