# Supplementary material for: An Isoform of the Oncogenic Splice Variant AIMP2-DX2 Detected by a Novel Monoclonal Antibody
Source: Biomolecules. 2020 May 27;10(6):820. doi: 10.3390/biom10060820 (PMC7356629; doi:10.3390/biom10060820)
Supplement: Supplementary file 1 [file biomolecules-10-00820-s001.pdf]

An isoform of the oncogenic splice variant AIMP2-DX2 detected by a novel monoclonal antibody

Dae Gyu Kim, Thi Thu Ha Nguyen, Nam Hoon Kwon, Junsik Sung, Semi Lim, Eun-Joo Kang, Jihye Lee, Woo Young Seo, Arum Kim, Yoon Soo Chang, Hyunbo Shim, and Sunghoon Kim

Supplemental Information

Table S1. Primers designed for amplification of variable regions.

Table S2. Primers used for RT-PCR.

Table S3. Primers designed for overlap extension PCR to join fragments into Fab.

Table S4. Cleavage sites in DX2 between 25–41 amino acids predicted by PeptideCutter program

Figure S1. Cloning process of Fab fragment generation.

Figure S2. ELISA with clone #324 and rH5 antibody.

Figure S3. Strategy for the identification of exon1/3 junction peptide of DX2 from the immunoprecipitated protein using rH5 antibody and subsequent mass spectrometry analysis.

Figure S4. Identification of DX2 isoform detected by H5.

Figure S5. Full images of western blot membranes in Figure 1.

Figure S6. Full images of western blot membranes in Figure 2.

Figure S7. Full images of western blot membranes in Figure 3.

Figure S8. Immunostaining of normal lung tissues with H5 mAb.

Supplemental Tables

Table S1. Primers designed for the amplification of rabbit variable domain genes.

Type	Name	Sequence
Heavy Chain	RVH1	GCC CAA CCA GCC ATG GCC CAG GAG CAG CTG AAG GAG
	RVH2	GCC CAA CCA GCC ATG GCC CAG GAG CAG CTG RTG GAG
	RVH3	GCC CAA CCA GCC ATG GCC CAG GAG CAG CTG GAG GAG TCC
	RVH4	GCC CAA CCA GCC ATG GCC CAG TCG STG GAG GAG TCC
	RVH5	GCC CAA CCA GCC ATG GCC CAG TCG GTG AAG GAG TCC
	RVH6	GCC CAA CCA GCC ATG GCC CAG CAG CTG GAG CAG TCC
Light Chain (kappa)	RVK1	TAA TTG GCC CAG GCG GCC GAC CCT ATG CTG ACC CAG
	RVK2	TAA TTG GCC CAG GCG GCC GAT GTC GTG ATG ACC CAG
	RVK3	TAA TTG GCC CAG GCG GCC GCA GCC GTG CTG ACC CAG
	RVK4	TAA TTG GCC CAG GCG GCC GCC ATC GAT ATG ACC CAG
	RVK5	TAA TTG GCC CAG GCG GCC GCC CAA GTG CTG ACC CAG
	RVK6	TAA TTG GCC CAG GCG GCC GCC CTT GTG ATG ACC CAG
	RVK7	TAA TTG GCC CAG GCG GCC GCT CAA GTG CTG ACC CAG
	RVK8	TAA TTG GCC CAG GCG GCC TAT GTC ATG ATG ACC CAG
Light Chain (Lambda)	RVL1	TAA TTG GCC CAG GCG GCC CAG CCT GCC CTC ACT CAG
	RVL2	TAA TTG GCC CAG GCG GCC TCC TAT GAG CTG ACA CAG
	RVL3	TAA TTG GCC CAG GCG GCC TCC TTC GTG CTG ACT CAG
	RVL4	TAA TTG GCC CAG GCG GCC CAG CCT GTG CTG ACT CAG
	RVL5	TAA TTG GCC CAG GCG GCC AGC GTT GTG TTC ACG CAG
	RVL6	TAA TTG GCC CAG GCG GCC CAG TTT GTG CTG ACT CAG
Heavy Chain	RJH-b	TGG GCC CTT GGT GGA GGC TGA RGA GAY GGT GAC CAG GGT
Light Chain (kappa)	RJK1-b	AGA TGG TGC AGC CAC AGT TCG TTT GAT TTC CAC ATT GGT
	RJK2-b	AGA TGG TGC AGC CAC AGT TCG TTY GAC SAC CAC CTY GGT
	RJK3-b	AGA TGG TGC AGC CAC AGT TCG TAG GAT CTC CAG CTC GGT
	RJK4-b	AGA TGG TGC AGC CAC AGT TCG TTT GAT YTC CAS CTT GGT
Light Chain (Lambda)	RJL-b	AGA TGG TGC AGC CAC AGT TCG GCC TGT GAC GGT CAG CTG GGT

RVH1~6, forward primers for V_H (variable heavy chain) amplification; RVK1~8, forward primers for Vk (variable kappa chain); RVL1~6, forward primers for V_L (variable lambda chain); RJH-b1, reverse primer for V_H; RJK1~4b, reverse primers for V_K; RJL-b, reverse primer for V_L.

Table S2. Primers used for RT-PCR.

Type	Primer combinations							
Heavy Chain	RVH1 RJH-b1	RVH2 RJH-b1	RVH3 RJH-b1	RVH4 RJH-b1	RVH5 RJH-b1	RVH6 RJH-b1		
Light Chain (kappa)	RVK1 RJK-b1	RVK2 RJK-b1	RVK3 RJK-b1	RVK4 RJK-b1	RVK5 RJK-b1	RVK6 RJK-b1	RVK7 RJK-b1	RVK8 RJK-b1
	RVK1 RJK-b2	RVK2 RJK-b2	RVK3 RJK-b2	RVK4 RJK-b2	RVK5 RJK-b2	RVK6 RJK-b2	RVK7 RJK-b2	RVK8 RJK-b2
	RVK1 RJK-b3	RVK2 RJK-b3	RVK3 RJK-b3	RVK4 RJK-b3	RVK5 RJK-b3	RVK6 RJK-b3	RVK7 RJK-b3	RVK8 RJK-b3
	RVK1 RJK-b4	RVK2 RJK-b4	RVK3 RJK-b4	RVK4 RJK-b4	RVK5 RJK-b4	RVK6 RJK-b4	RVK7 RJK-b4	RVK8 RJK-b4
Light Chain (lambda)	RVL1 RJL-b1	RVL2 RJL-b1	RVL3 RJL-b1	RVL4 RJL-b1	RVL5 RJL-b1	RVL6 RJL-b1		

Forward and reverse primers presented in Supplementary Table 1 were paired to generate 44 combinations to amplify heavy and light chain variable domains.

Table S3. Primers designed for overlap extension PCR to join fragments into Fab.

Name	Sequence
HIgGH1-f	GCC TCC ACC AAG GGC CCA
Dpseq	AGA AGC GTA GTC CGG AAC G
HKC-f	ACT GTG GCT GCA CCA TCT G'
Lead-b	GGC CAT GGC TGG TTG GGC
LeadVH	GCC CAA CCA GCC ATG GCC
RSC-SF	TAA TTG GCC CAG GCG GCC'

Table S4. Cleavage sites in DX2 between amino acid positions 25–41, predicted by PeptideCutter program.

Position of cleavage site	Cleaving enzyme(s)	Resulting cleaved residue
26	Arg-C proteinase	R (P1)
	Clostripain	
	Trypsin	
27	Pepsin (pH1.3)	L (P1)
	Pepsin (pH>2)	
	Proteinase K	
29	Thermolysin	N (P1')
30	Proteinase K	V (P1)
31	Chymotrypsin-low specificity	H (P1)
33	Arg-C proteinase	R (P1)
	Clostripain	
	Trypsin	
34	Pepsin (pH>2)	S (P1)
35	Chymotrypsin-high specificity	Y (P1)
	Chymotrypsin-low specificity	
	Proteinase K	
38	Proteinase K	A (P1)
40	Thermolysin	G (P1')

Potential cleavage sites of DX2 N-terminus predicted by PeptideCutter (https://web.expasy.org/peptide_cutter). Position of cleavage site shows the amino acid number of DX2 protein that could be recognized by specific enzymes. Cleavage might occur right after the amino acid (P1), except thermolysin which cleaves the peptide bond right before the specificity residue (P1').

Supplemental Figures.

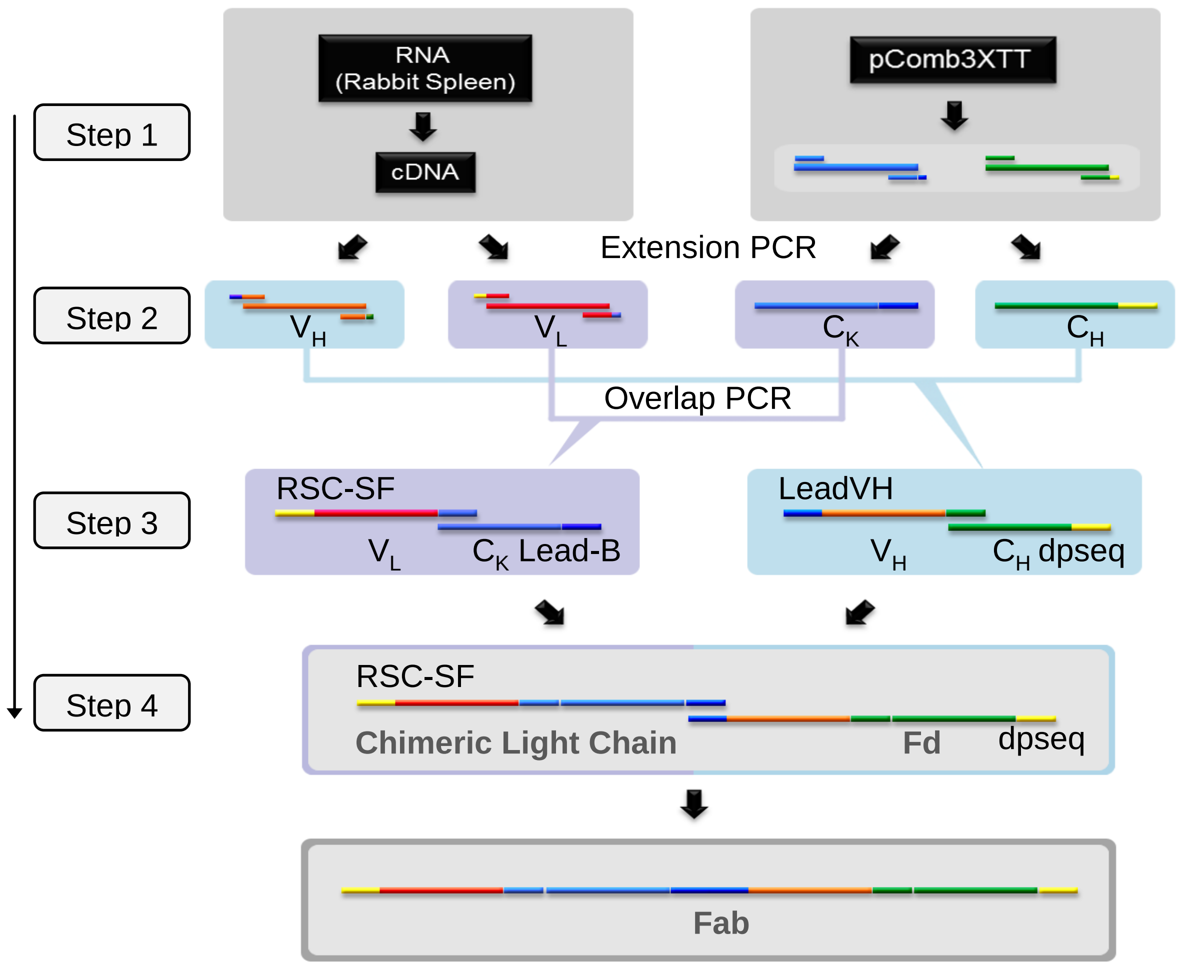

Figure S1. Cloning process of Fab fragment generation. Overall schematic map of antibody fragment generation by overlap extension PCR is shown. The first step is to synthesize cDNA from mRNA extracted from the spleen of the immunized rabbit and to generate constant light and heavy chains (C_k and C_{H1}) from pComb3X-TT phage vector containing a Fab gene. The second step is to amplify V_H/V_L from cDNA synthesized from the extracted mRNA. For the third step, V_L and C_k were fused using RSC-SF/Lead-B primers, and V_H and C_{H1} were fused using LeadVH/dpseq primers by overlap extension PCR. Last step is to combine the chimeric light chain and the heavy chain Fd into full length Fab fragment.

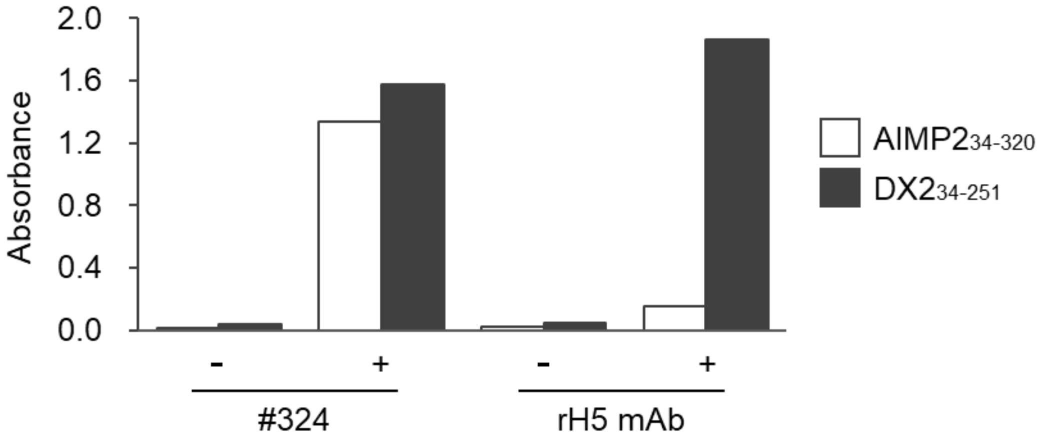

Figure S2. ELISA of clone #324 and rH5 antibody. Specificities of clone #324 and rH5 mAb to $DX2_{34-251}$ and $AIMP2_{34-320}$ were compared using ELISA.

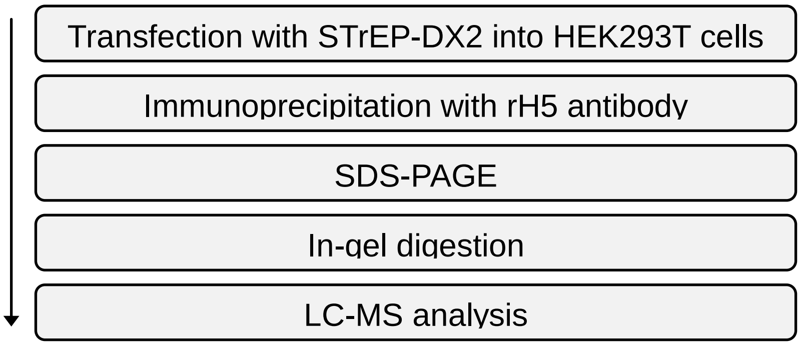

Figure S3. Strategy for the identification of exon1/3 junction peptide of DX2 from the immunoprecipitated protein using rH5 antibody and subsequent mass spectrometry analysis. HEK293T cells expressing Strep-DX2 were immunoprecipitated using rH5 antibody and the immunoprecipitate was subjected to SDS-PAGE and LC-MS analysis.

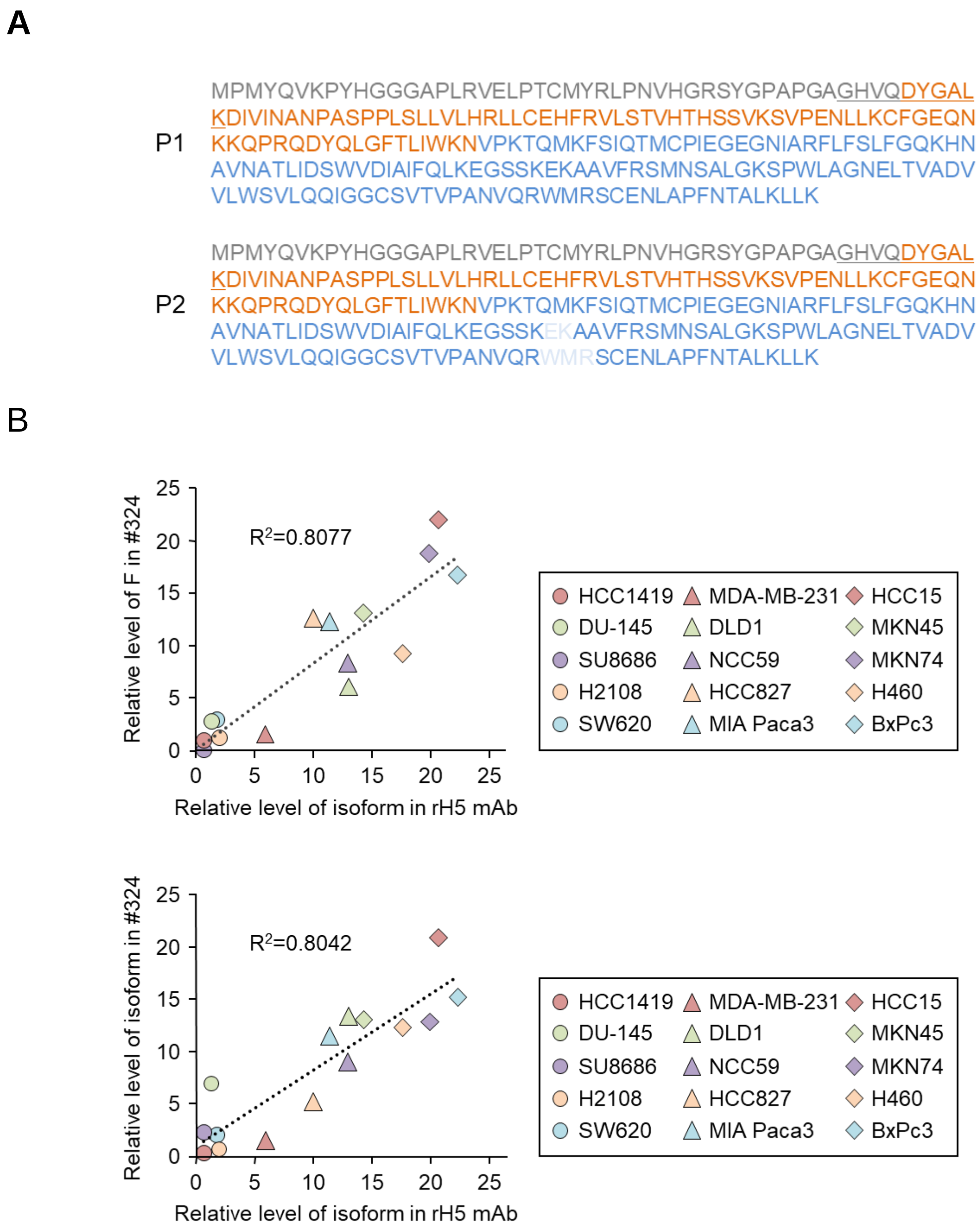

Figure S4. Identification of DX2 isoform detected by H5. (**A**) Identified sequence of DX2 proteins P1 and P2 by mass analysis. Light grey letters indicate unidentified sequences. P1 and P2 are the DX2-F protein before and after trypsin digestion, respectively, as shown in Fig. 3B. (**B**) The quantitated levels of DX2 isoform detected by rH5 mAb and those of DX2-F (upper) or DX2 isoform (lower) recognized by clone #324 in various cancer cell lines were presented with R^2 (Square of the person correlation coefficient) value.

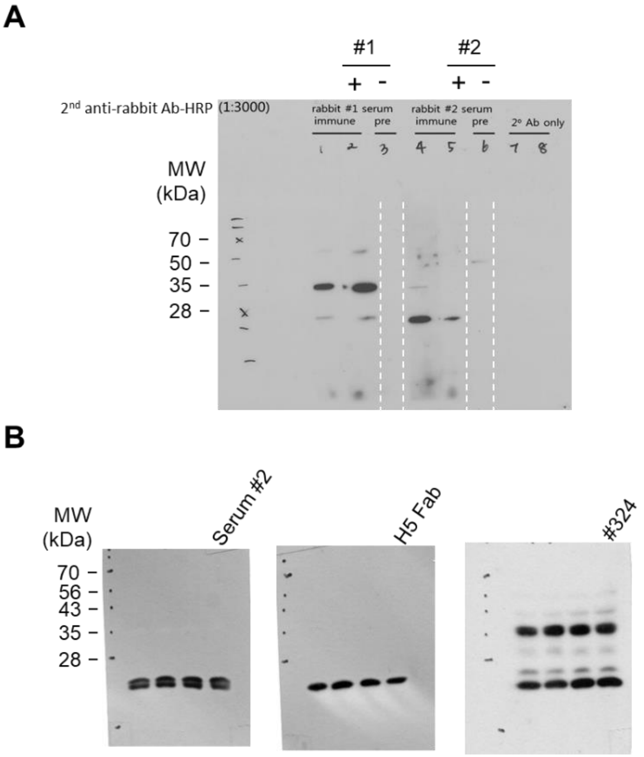

Figure S5. Full images of western blot membranes for Figure 1. (**A**) Western blot analysis with rabbit sera. Lanes 2 and 3 and lanes 5 and 6 were cropped and presented in Figure 1B. The membrane was cut into pieces and separately incubated with rabbit #1 serum (lanes 1 and 2), rabbit #2 serum (lanes 4 and 5), rabbit #1 serum before immunization (lane 3), and rabbit #2 serum before immunization (lane 6). After incubation with each primary serum, the membrane pieces were incubated with secondary anti-rabbit antibody conjugated with HRP. (**B**) Western blot results with rabbit serum #2 (left), H5 Fab (middle), and #324 (right). Each membrane contains quadruplicates, each far right lane of which is presented in Figure 1C.

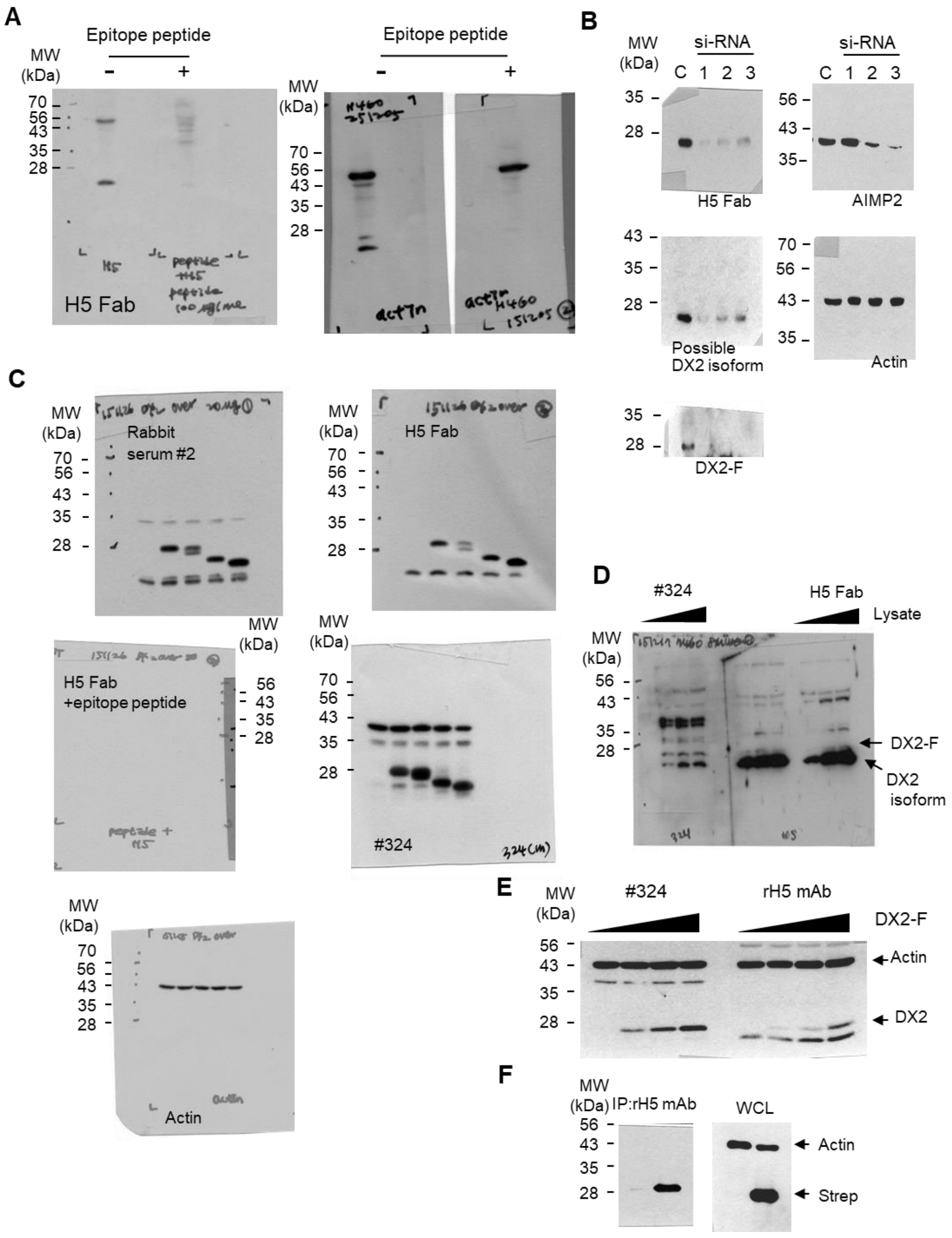

Figure S6. Full images of western blot membranes for Figure 2. (**A**) Western blot analysis with (+) or without (-) epitope peptide. (**B**) Western blot results with si-RNA treatment. The membrane was cut into pieces to detect different protein simultaneously. (**C**) Western blot analysis of Strep-DX2 fragments. (**D**) Western blot analysis using 8M urea sample buffer. (**E**) Western blot analysis of tag-free DX2 overexpression with #324 and rH5. (**F**) Western blot analysis for the immunoprecipitation.

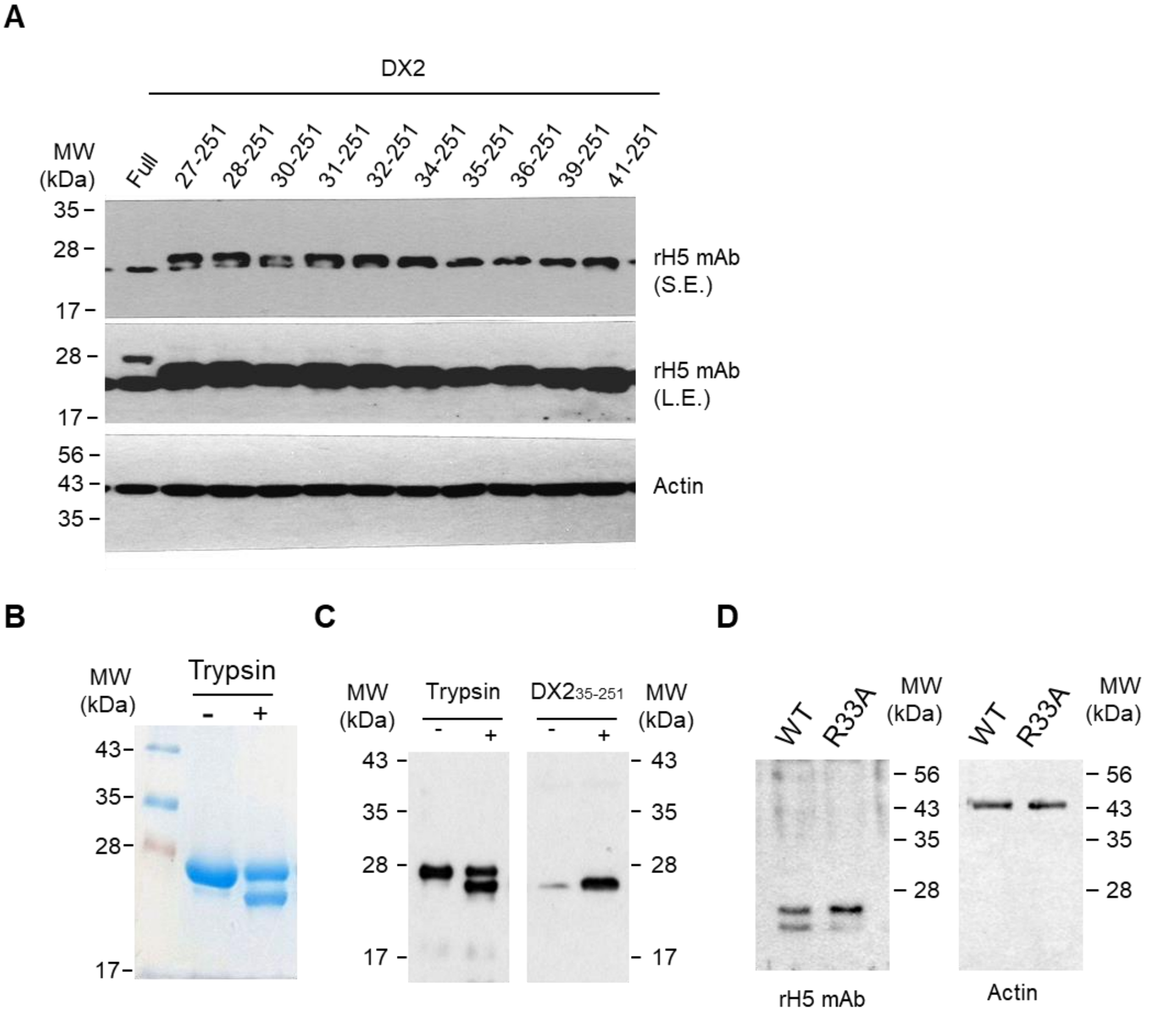

Figure S7. Full images of western blot membranes for Figure 3. (**A**) Western blot analysis of overexpressed tag-free DX2 fragments. (**B**) Coomassie stained gel image of limited proteolysis. (**C**) Western blot analysis for DX2 digested with trypsin and HEK293T cell lysates expressing DX2$_{35-251}$. (**D**) Western blot analysis of DX2 wild type and R33A mutant.

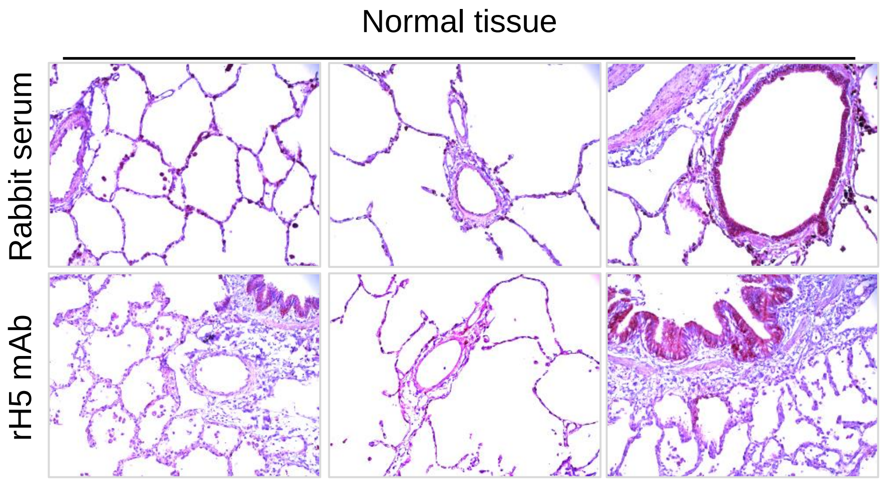

Figure S8. Immunostaining of normal lung tissues with H5 mAb. Levels of DX2 in human normal lung were determined by immunohistochemistry staining using #2 rabbit serum and rH5 antibody (x200).
